# Transient Receptor Potential Channels Encode Volatile Chemicals Sensed by Rat Trigeminal Ganglion Neurons

**DOI:** 10.1371/journal.pone.0077998

**Published:** 2013-10-21

**Authors:** Matthias Lübbert, Jessica Kyereme, Nicole Schöbel, Leopoldo Beltrán, Christian Horst Wetzel, Hanns Hatt

**Affiliations:** 1 Department of Cell Physiology, Ruhr University Bochum, Bochum, Germany; 2 Leibniz Research Centre for Working Environment and Human Factors, University of Dortmund, Dortmund, Germany; 3 Department of Psychiatry and Psychotherapy, University of Regensburg, Regensburg, Germany; German Institute of Human Nutrition Potsdam-Rehbruecke, Germany

## Abstract

Primary sensory afferents of the dorsal root and trigeminal ganglia constantly transmit sensory information depicting the individual’s physical and chemical environment to higher brain regions. Beyond the typical trigeminal stimuli (e.g. irritants), environmental stimuli comprise a plethora of volatile chemicals with olfactory components (odorants). In spite of a complete loss of their sense of smell, anosmic patients may retain the ability to roughly discriminate between different volatile compounds. While the detailed mechanisms remain elusive, sensory structures belonging to the trigeminal system seem to be responsible for this phenomenon. In order to gain a better understanding of the mechanisms underlying the activation of the trigeminal system by volatile chemicals, we investigated odorant-induced membrane potential changes in cultured rat trigeminal neurons induced by the odorants vanillin, heliotropyl acetone, helional, and geraniol. We observed the dose-dependent depolarization of trigeminal neurons upon application of these substances occurring in a stimulus-specific manner and could show that distinct neuronal populations respond to different odorants. Using specific antagonists, we found evidence that TRPA1, TRPM8, and/or TRPV1 contribute to the activation. In order to further test this hypothesis, we used recombinantly expressed rat and human variants of these channels to investigate whether they are indeed activated by the odorants tested. We additionally found that the odorants dose-dependently inhibit two-pore potassium channels TASK1 and TASK3 heterologously expressed In *Xenopus laevis* oocytes. We suggest that the capability of various odorants to activate different TRP channels and to inhibit potassium channels causes neuronal depolarization and activation of distinct subpopulations of trigeminal sensory neurons, forming the basis for a specific representation of volatile chemicals in the trigeminal ganglia.

## Introduction

All sensory systems are based on specialized cells and provide a constant flow of information from the periphery to central structures. Somatosensory neurons are located in sensory ganglia such as the dorsal root ganglia (DRG) or the analog structures of the head, the trigeminal ganglia (TG) [1]. Neurons of the TG extend their peripheral terminals to the facial skin, the mucosae, and the meninges. Here, they function as chemo-, mechano-, and thermosensors, as well as nociceptors [2]–[4]. The trigeminal system contributes to overall chemosensation and interestingly, most if not all odorants in higher concentrations stimulate sensory neurons belonging to the trigeminal system [5]–[8]. The remarkably broad sensory capacity of TG neurons is fundamentally based on the expression of various receptors, such as members of the transient receptor potential (TRP) family, two-pore potassium (K_2_P) channels, or acid-sensing ion channels [9], [10]. Among these receptors, TRPV1, TRPM8, and TRPA1, are highly expressed in C- and Aδ-fibers of the DRG and TG [11]–[14].

TRPV1 is activated by a variety of physical and chemical stimuli, such as heat, low pH, exogenous (e.g. capsaicin) or endogenous (e.g. anandamide) vanilloid ligands, polyunsaturated fatty acids [15], [16], and divalent cations like Mg^2+^, Ca^2+^, Cu^2+^, or Ni^2+^ [17]–[19]. TRPM8 is activated by cool to noxiously cold temperatures, natural chemical ligands such as menthol, eucalyptol, and linalool, or synthetic chemical ligands like icilin [20], [21]. Both, TRPV1 and TRPM8 are furthermore activated by membrane depolarization [22]. Stimuli activating TRPA1 include electrophilic agents such as isothiocyanates, α,-β-unsaturated aldehydes (e.g. cinnamaldehyde), cannabinoids (D9-tetrahydrocannabinol), nicotine, Ca^2+^-ions and noxiously cold temperatures [15], [23]. Furthermore, all three channels are weakly activated by higher concentrations of the odorants geraniol and citral [24]–[26]. Besides its well described somatosensory functions such as thermosensation or as an alerting system that detects potentially harmful stimuli, the trigeminal system is able to discriminate different volatile chemicals. In this context, it was shown that anosmics, who have lost fine odor discriminative skills, retain the ability to distinguish between different odor categories [27]. For some compounds, this selectivity is even sufficient to discriminate between different stereoisomers (e.g. (+)- and (−)-nicotine) [28]. Although several studies addressed the question how volatile chemicals are represented in higher brain regions [29]–[32], nearly nothing is known about the impact of the TG on the representation of different volatiles in the brain. Recently one study described stimulus-specific activity patterns at the level of the TG *in vivo* [33]. The authors described trigeminal activation by the odorants citral and vanillin, the latter being a stimulus that previously was considered to exclusively activate the olfactory system [7], [34], [35]. Little is known about the molecular mechanisms triggering stimulus-specific activity patterns in the TG and about the mechanisms through which odorants activate trigeminal afferents. In summary, some odorants are known to stimulate the trigeminal system at concentrations that are generally well above those required for olfactory reception [36]. The mechanisms underlying the compound-induced activation of individual TG neurons or the generation of the described activity patterns remain elusive.

The aim of the present study was therefore to identify receptors expressed in rat TG neurons that are modulated by different odorants. We performed Ca^2+^ imaging and electrophysiological recordings to screen eight different odorants with respect to their potential to depolarize TG neurons *in vitro*. Dose-dependent depolarization induced by vanillin, heliotropyl acetone (HTPA), helional, and geraniol were mediated by TRPV1, TRPA1, and TRPM8. The K_2_P channels TASK1 and TASK3 may further contribute to the signals, as they were inhibited by the odorants. Based on our data, we suggest that different TRP channel combinations in distinct TG neuron populations contribute to the stimulus-specific representations of distinct odorants in the TG. This may underlie the previously observed activity patterns in the TG and contribute to the discriminative abilities mediated by the trigeminal system.

## Materials and Methods

### Animals

Experiments were conducted according to the Animal Protection Law of the Federal Republic of Germany (TierSchG BGBI.I,S.1206, revision 2006) and European Communities Council directive regarding care and use of animals for experimental procedures (86/6009/EEC). Approval by the Ethics Committee or the previous announcement for this kind of experiments is not necessary in accordance with the Animal Protection Law of the Federal Republic of Germany (§ 4 of the TierSchG). The amount of animals used was reported to the corresponding authority. All efforts were made to minimize the number of animals and their suffering. Trigeminal ganglia were removed from rats after decapitation by appropriately trained staff with approval of LANUV NRW (Landesamt für Umwelt, Natur und Verbraucherschutz Nordrhein Westfalen, Düsseldorf). All experiments were performed using wistar rats of both genders at P (postnatal day) 1−5. Water and food were offered *ad libitum*.

### Primary culture of trigeminal ganglion neurons

Cultures of TG neurons were obtained as previously described [37]. In brief, Wistar rats (P1-5) were decapitated, the skull was opened, and the brain was removed. After dissection of the paired TG, they were washed in PBS^+/+^ and gathered in minimal essential medium (MEM, Invitrogen, Germany) containing 0.025% collagenase (type IA, Sigma Aldrich, USA). Ganglia were minced and incubated for 45 min under humidified conditions (37°C, 95% air, 5% CO_2_). After digestion, tissue was triturated with fire polished glass pipettes of decreasing tip-diameter and the cell suspension was centrifuged (4 min, 157 rcf). The pellet was resuspended in Dulbecco’s modified eagle medium F-12 (DMEM/F12, GlutaMax, Invitrogen, Germany) supplemented with 10% fetal bovine serum (FBS) and 100U/ml penicillin and 100 µg/ml streptomycin (pen/strep). No additional additives such as neurotrophins were added to the medium. Afterwards, 50 µl of the cell suspension was plated in 0.01% poly-L-lysine coated 35 mm cell culture petri dishes (Sarstedt, Germany) for patch-clamp recordings or on lysine-coated glass coverslips (30 mm) for Ca^2+^ imaging experiments. After one hour, 2 ml DMEM/F12 (+10% FBS, 1% pen/strep) were added to the attached cells which were then kept under humidified conditions (37°C, 95% air, 5% CO_2_) until experiments were performed 1−3 days after preparation.

### Cultivation of cell lines

CHO cells were cultured in T75-cell culture flasks in MEM containing Earl`s salts and L-glutamine (Invitrogen, Germany), containing 10% FBS and pen/strep under humidified conditions (37°C, 95% air, 5% CO_2_).

### Transient transfection of CHO cells

For transient expression of the recombinant rTRPV1, rTRPM8, or rTRPA1 channel proteins, we used the mammalian expression vector pcDNA3 (Invitrogen, Germany). The plasmids carried the complete coding sequence of the respective protein. Semiconfluent cells were transiently transfected (1 µM cDNA per dish) in 35-mm cell culture petri dishes (Sarstedt, Germany), using the CaP-precipitation method as previously described [19]. Green fluorescent protein (GFP) was used as a transfection marker. Recordings were performed within ∼18−48 h after transfection. Wild type rTRPV1, hTRPV1, hTRPM8 and rTRPM8 plamids cDNAs kindly provided by D. Julius, rTRPA1 cDNA by D. Clapham, and the hTRPA1 cDNA from Grünenthal (Germany).

### Patch-clamp recordings

Patch pipettes were pulled from borosilicate glass capillaries (1.17×1.50×100 mm, Science Products, Germany) and were fire polished to 2.5−7 MΩ tip resistance using a horizontal puller (Zeitz Instruments, Germany). We used two different intracellular buffers which were either based on K^+^ (140 mM KCl, 0.1 mM CaCl_2_, 1 mM MgCl_2_, 5 mM EGTA, 10 mM HEPES, and 2 mM ATP, pH 7.4) or on Cs^+^ (140 mM CsCl, 2 mM MgCl_2_, 1 mM CaCl_2_, 11 mM EGTA, 2 mM HEPES, and 2 mM ATP, pH 7.4). If not otherwise stated, the Na^+^-based extra- and the K^+^-based intracellular buffer were used for recordings which were performed at room temperature (RT). We used an EPC7 amplifier (HEKA, Germany). Capacity and serial resistance was adjusted manually. Data were acquired using the Pulse software (HEKA, Germany). Between stimulus applications, cells were constantly perfused with extracellular buffer (Na^+^-based: 140 mM NaCl, 5 mM KCl, 2 mM CaCl_2_, 1 mM MgCl­_2_, 10 mM HEPES, pH 7.4; Cs^+^-based: 145 mM CsCl, 2 mM CaCl_2_, 1 mM MgCl_2_, 10 mM HEPES, pH 7.4; Ca^2+^-free: 140 mM NaCl, 10 mM HEPES, 5 mM EDTA, pH 7.4). Stimuli were diluted in the respective extracellular buffer and were applied to the investigated cell via a custom-made manifold superfusion device.

Prior to recordings from TG neurons, cells were depolarized by a single voltage ramp from −100 to +100 mV under voltage-clamp settings (0.286 mV/ms). Further recordings were performed exclusively on cells exhibiting voltage-activated currents upon membrane depolarization. Similar ramps were applied with an interval of 2s during all voltage clamp recordings (VC recordings). No currents were injected during current clamp recordings (CC recordings). We observed an averaged resting membrane potential (RMP) of −59.1 −52.2/−66 mV throughout all CC recordings performed (n = 479 neurons).

### Calcium imaging experiments

Prior to experiments, cells were incubated with the Ca^2+^-sensitive dye Fura-2/AM (Invitrogen, Germany) at 37°C, for 45−60 min. For measurements at RT, they were placed in a recording chamber from inert steel and were mounted on an inverted microscope (Axiovert 200M, Zeiss, Germany) which was equipped with a fluorescent-optimized 20-fold Zeiss UPlanApo (20×/0.75) objective. Fura-2 was excited intermittently for 100 ms at wavelengths of 340 nm and 380 nm (Lambda DG4 (Sutter Instrument Company, USA) connected to a Uniblitz Vmm-Di shutter driver and a Voltcraft condenser) at 1 Hz. Emitted light was detected at 510 nm via a Zeiss Axiocam MRM CCD-camera (Zeiss, Germany). Changes of [Ca^2+^]_i_ were monitored as the ratio of the 510 nm emission for both excitation wavelength (f_340_/f_380_). Data were acquired using the Slide-Book software (3I-Imaging, Germany). Between stimulus applications, cells were perfused with extracellular buffer (140 mM NaCl, 5 mM KCl, 2 mM CaCl_2_, 1 mM MgCl­_2_, 10 mM HEPES, adjusted to pH = 7.4 and 300 mosmol/l using glucose).

### Two electrode voltage clamp recordings

Two electrode VC recordings were performed as previously described [38], [39]. In short, electrodes (0.1−0.5 MΩ) were made using a Kopf vertical micropipette puller (Kopf instruments, USA) and filled with 3 M KCl. *Xenopus laevis* oocytes were placed in a chamber and perfused with Ringer-solution (115 mM NaCl, 2.5 mM KCl, 1.8 mM CaCl_2_, 10 mM HEPES; pH = 7.2). Currents were recorded using a two-electrode voltage-clamp amplifier (TURBO TEC-03, npi, Germany) and analyzed by using the pCLAMP software (Axon Instruments, USA). During recordings, we used voltage ramps from −100 to +50 mV (0.21 mV/ms) followed by a 300 ms constant at +50 mV with a 2s interval. In order to evaluate the effect of an odorant at a given concentration, we took the average of the current registered at the final 30 ms of the +50 mV period in the three ramps exhibiting maximal responses to the administered substances. These were then normalized relative to the averaged currents monitored prior to stimulus-application. All experiments were performed at RT 24-72 h after cRNA injection. cRNAs were prepared using standard molecular biology procedures [40]. Receptor cDNA cloned into pEXO was kindly provided by Dr. Amanda Patel.

### Analysis of electrophysiological and calcium imaging data

Electrophysiological data were analyzed using the Pulse software (HEKA, Deutschland), and Ca^2+^ imaging data with the Slide-Book software (3I-Imaging, Germany). For further analysis we used IgorPro (Wavemetrics, USA), MATLAB (MathWorks, USA), Origin (Originlab Corp., USA) and Microsoft Excel (Microsoft Corp., USA). Membrane potential changes and recorded currents were regarded as statistically significant when exceeding four times the standard deviation of resting membrane potential fluctuations or baseline fluctuations. Since not all datasets were normally distributed (χ^2^-test), significance was tested by the Wilcoxon-signed-rank test (paired data) or the Man-Whitney-U-Test (unpaired data). Therefore, all data are presented as median +3^rd^/−1^st^ quartile in tables. All statistically significant changes are indicated by asterisks within figures. If not otherwise stated, data are depicted as box plot diagrams with: Red line: median; white square: mean; upper/lower edges of the gray boxes: 1^st^ and 3^rd^ quartile; whiskers: 5^th^ and 95^th^ percentile; x: outliers.

### Solutions and chemicals

Capsaicin (cap), menthol (men), allyl isothiocyanate (AITC), capsazepine, and HC-030031 were prepared as concentrated stock solutions in DMSO, odorant-solutions (2-phenylethanol (PEE), sandalore, sandranol, yavanol, vanillin, HTPA, geraniol, helional) were always prepared freshly prior to the experiments. Capsaicin, menthol, AITC, HC-030031, BCTC **(**4-(3-Chloro-2-pyridinyl)-N-[4-(1,1-dimethylethyl)phenyl]-1-piperazinecarboxamide), and capsa-zepine were purchased from Sigma Aldrich (USA). With exception of vanillin (Sigma Aldrich, USA), all odorants were kindly provided by Symrise (Germany).

## Results

### Different odorants depolarize cultured TG neurons

A recent study reported that nasal administration of different volatile chemicals (e.g. vanillin) evoked stimulus-specific activity patterns in the TG of anesthetized rats [33]. Due to incomplete knowledge about the mechanisms underlying these activations and the receptors mediating the detection of these substances, we screened 8 different odorants for their effects on the membrane potential of cultured TG neurons.

Therefore, we performed whole-cell current clamp recordings (CC recordings) from cultured TG neurons that we challenged either with vanillin, sandalore, 2-Phenylethanol (PEE), helional, or geraniol. All substances used were previously shown to trigger activity in the TG upon nasal administration *in vivo* (unpublished data). Additionally, we tested substances that are structurally similar to sandalore, namely sandranol and javanol, as well as HTPA which activates the same olfactory receptor (OR17-40) as does helional [40]. Substances were administered at concentrations of 1 mM for 8 s. Stimulus-induced membrane potential changes were regarded as statistically significant when exceeding the standard deviation of resting membrane potential fluctuations by a factor of four. A total amount of n = 479 neurons was investigated in CC recordings in the current study. Averaged, these neurons featured a resting membrane potential (RMP) of −59.1 −52.2/−66 mV.

**Figure 1:**
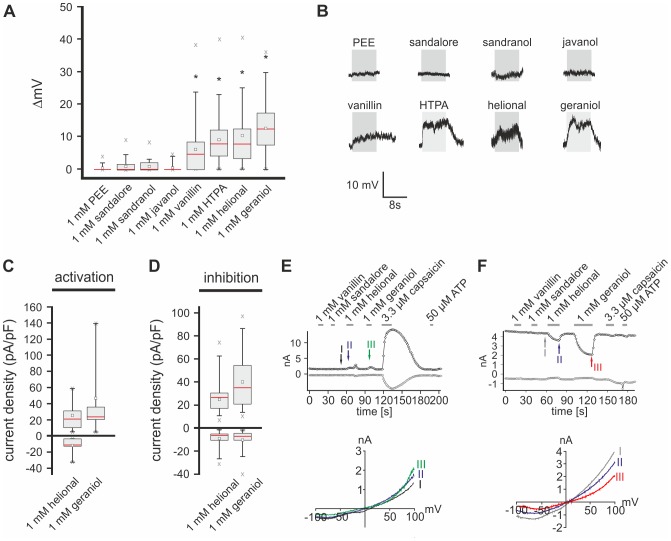
Different odorants activate cultured TG neurons. **A:**Box plot diagram depicting membrane potential changes in TG neurons challenged with PEE (n = 31), sandalore (n = 31), sandranol (n = 31), javanol (n = 31), vanillin (n = 68), HTPA (n = 96), helional (n = 129) and geraniol (n = 108). Significant depolarization was evoked by vanillin, HTPA, helional, and geraniol. Amplitudes can be derived from table 1. **B:** Exemplary whole-cell CC recordings of TG neurons upon stimulation with PEE, sandalore, sandranol, javanol, vanillin, HTPA, helional, and geraniol (1 mM each). RMP: PEE: −58 mV; sandalore: −62 mV; sandranol: −62 mV; javanol: −55 mV; vanillin: −60 mV; HTPA: −61 mV; helional: −58 mV; geraniol: −55 mV. **C,D:** Box plot diagrams depicting densities (pA/pF) of activated (C) and inhibited (D) currents during whole-cell VC recordings from TG neurons upon stimulation with helional (n = 33) and geraniol (n = 32). Outward currents recorded at +100 mV are depicted by upward facing bars, inward currents recorded at −100 mV are depicted by downward facing bars. Detailed values can be derived from table 2. **E,F:** Whole-cell VC recordings from TG neurons challenged with vanillin, sandalore, helional, geraniol, cap, and ATP depicting odorant-induced activation (E) and inhibition (F) of currents. Current amplitudes at +100 (*black*) and −100 mV (*gray*) are plotted vs. time (*above*). Stimulus application is indicated by the gray bars. Colored arrows and roman numerals assign to individual voltage ramps depicted in the corresponding IV-plots (*below*).

Administration of vanillin, HTPA, helional, and geraniol depolarized the membrane potential of TG neurons whereas PEE, sandalore, sandranol, and javanol did not (fig. 1A and B, table 1). Next, we were interested in the efficacy of these odorants to activate TG neurons. We used Ca^2+^ imaging to establish dose-effect relations and determined an EC_50_ of 3.79 ± 0.18 mM for HTPA with a threshold of 0.1 to 1 mM (fig. S1). Activation thresholds for helional with an EC_50_ of 0.31 ± 0.39 mM and geraniol with an EC_50_ of 0.37 ± 0.66 mM were between 0.01 and 0.1 mM (fig. S1). Testing of saturating concentrations of vanillin and thereby determination of a corresponding EC_50_ was prevented by its limited solubility in aqueous solutions. Nevertheless, vanillin induced a concentration-dependent increase of intracellular Ca^2+^ with an activation threshold between 0.1 and 1 mM (fig. S1).

**Table 1:** Averaged amplitudes of vanillin-, HTPA-, helional-, and geraniol-evoked depolariza-tions of cultured TG neurons.

	vanillin (n = 68)	HTPA (n = 79)	helional (n = 96)	geraniol (n = 75)
**Δ membrane potential [mV]**	4.8+8.35/−0	8+12.15/−4	7.8+12.9/−3.7	12+17.2/−7.4

Taken together, we can show that vanillin, HTPA, helional, and geraniol depolarize cultured TG neurons in a concentration-dependent manner whereas sandalore, sandranol, javanol, and PEE caused no measureable effect *in vitro*.

### Odorant-application alters the Cs^+^-conductance in TG neurons

Geraniol was previously shown to activate TRPV1, TRPA1, and TRPM8, which are highly expressed in TG neurons [11], [12], [41]. Therefore it is not surprising that geraniol causes depolarization of the membrane potential in these cells. However, nothing is known about potential receptors for helional and HTPA. Although vanillin was previously shown to activate TRPV3 at high concentrations [42], [43], the concentrations used in our study are considerably lower. Therefore, we hypothesize that other channels may be modulated by this substance.

Several odorants have been shown to inhibit delayed rectifier potassium channels [44]. To test for an involvement of these channels, we performed whole-cell voltage clamp recordings (VC recordings) using Cs^+^-based intra- and extracellular buffers in order to eliminate any K^+^-based currents. Additionally, this may help to isolate possible other receptors responsible for the observed odorant-evoked depolarization. At the end of every recording, cells were challenged with 3.3 µM capsaicin (cap) to test for functional TRPV1 expression, indicative of nociceptive neurons [11], [45], [46]. Application of helional induced significant outward and inward currents in 27.3% of the neurons tested, whereas currents elicited by geraniol occurred mainly in outward direction in 25% of the cells tested (fig. 1C and E, table 2). E_rev_ of helional- and geraniol-induced currents were not statistically different from those evoked by capsaicin (fig. 1E, table 3).

**Table 2:** Activated currents expressed as current densities in distinct TG neuron populations.

	helional (n = 33)		geraniol (n = 32)	
	in (−100 mV)	out (+100 mV)	in (−100 mV)	out (+100 mV)
**current densities [pA/pF]**	−9.72 +(−5.01)/− (−13.21)	22.1 +32.65/−10.65	no currents detected	26.78 +54.7/−23.59

In: inwardly directed currents at −100 mV; out: outwardly directed currents at +100 mV.

**Table 3:** Reversal potentials (E_rev_) of helional-, geraniol-, and capsaicin-evoked Cs^+^-based currents.

	helional	geraniol	capsaicin
**reversal potential (E_rev_) [mV]**	3.49 +4.85/−0.07	−1.17 +0/−4.27	0.5 +5.816/−0

Helional inhibited background currents in inward direction in 63.6% and in outward direction in 27.3% of neurons tested, while application of geraniol inhibited inward currents in 68.8% and outward currents in 75% of the investigated cells. Administration of vanillin and sandalore caused no detectable effect under the given conditions (fig. 1D and F; n = 17 each).

In summary, we could discriminate two populations of TG neurons in which odorants either increased or decreased membrane conductance under the given ionic conditions. Since we were mainly interested in the mechanisms underlying the observed depolarization of TG neurons, further experiments focused on possible receptors and ion channels whose modulation could depolarize the membrane potential of these cells.

### Different populations of odorant-sensitive TG neurons respond to capsaicin, allyl isothiocyanate, or menthol

As shown above, no differences were observed among cap- and odorant-evoked reversal potentials (E_rev_ = ∼0 mV). Since this is a typical feature of channels which unselectively conduct cations, such channels (among others) may contribute to the odorant-evoked currents recorded in TG neurons. A possible candidate represents TRPV1, since 63.3% of the helional- and geraniol-sensitive neurons were also sensitive for cap. Similar conductivities for cations are featured by TRPA1 and TRPM8 [47], which are also expressed in several populations of nociceptive afferents [10], [48].

**Figure 2:**
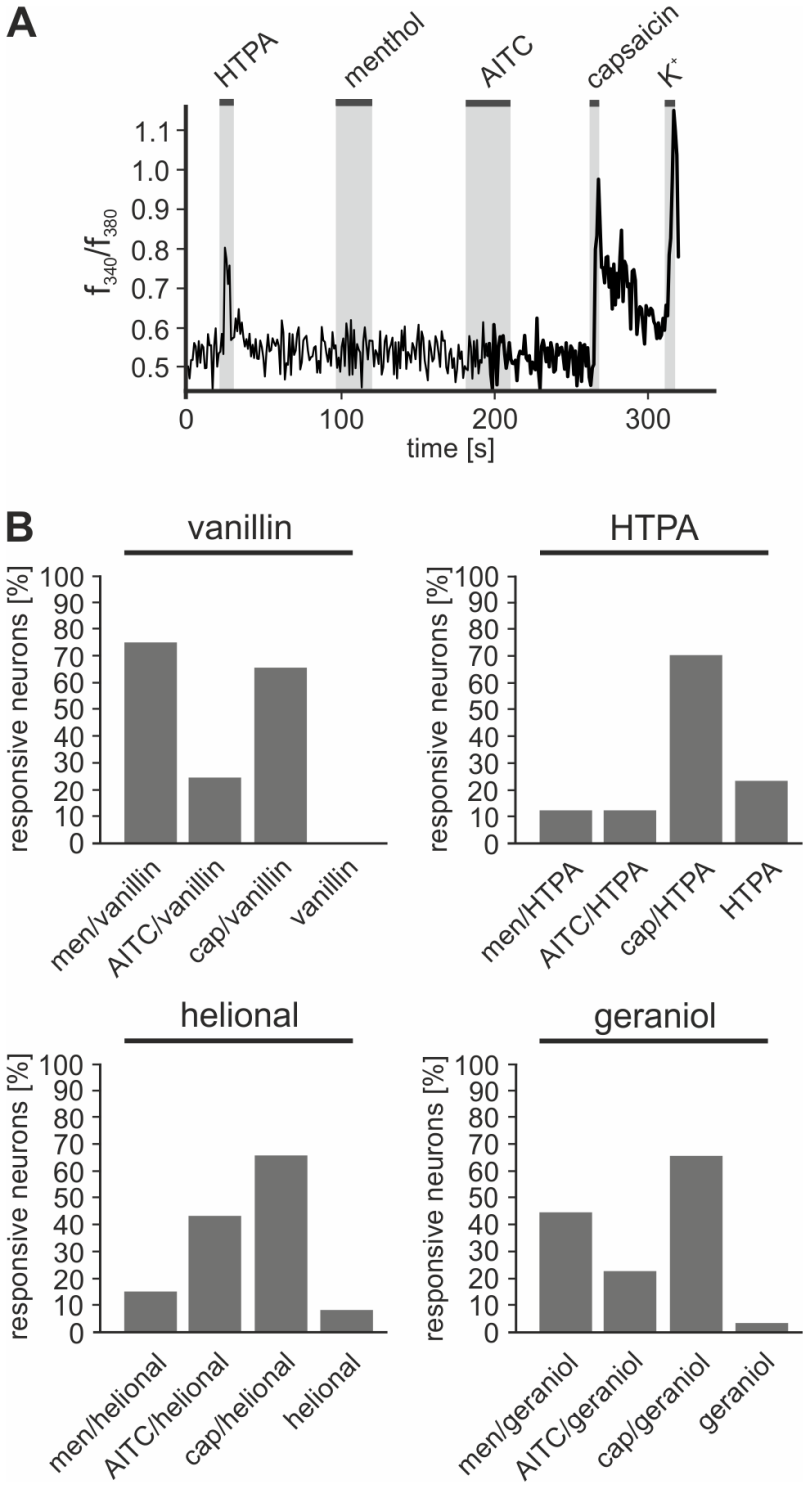
High numbers of TG neurons respond to odorants and TRP agonists. **A:** Ca^2+^ imaging recording of fura2/AM-loaded TG neurons. Cells were challenged with 1 mM HTPA, 50 µM AITC, 300 µM men, and 3.3 µM cap. Stimulus-application is indicated by gray bars. **B:** Bar charts depicting the overlap of responses to the odorants vanillin, HTPA, helional, or geraniol (1 mM each) and to the TRP-agonists cap (3.3 µM), men (300 µM), and AITC (50 µM).

In order to investigate if these TRP-channels are expressed in odorant-sensitive TG neurons and to identify different odorant-sensitive populations of TG neurons, we challenged Fura-2-AM-loaded TG neurons during Ca^2+^ imaging measurements with either one of the odorants vanillin, HTPA, helional, or geraniol (1 mM respectively), and either the TRPM8 agonist menthol (men; 300 µM), the TRPV1 agonist cap (3.3 µM) or the TRPA1 agonist AITC (50 µM). Substances were administered sequentially for 5−30 s with an interval of 60 s (fig. 2A).

Among 1159 TG neurons tested, 52.1% were sensitive for cap, 11.2% for AITC, and 10.8% for men. A total of 419 neurons were also stimulated with helional, which increased the intracellular Ca^2+^ level in 22.9% of investigated cells. Among these 22.9%, 66.7% were also sensitive for cap, 42.7% for AITC, 15.6% for men, and further 9.4% exclusively for helional. Geraniol application increased the intracellular Ca^2+^-level in 10.7% out of 458 neurons, of which 67.3% were also sensitive for cap, 44.2% for men, and 23.1% for AITC. Further 3.9% exclusively responded to geraniol. Out of the 129 cells that were challenged with HTPA, a total of 13.2% displayed responses to this compound. 70.6% of the HTPA sensitive cells were also sensitive to cap, 11.8% to AITC, 11.8% to men, while 23.5% of HTPA-sensitive cells lacked any responses to the TRP agonists. Sensitivity for vanillin was not observed in cap-, men-, or AITC-insensitive cells. 7.8% of the 153 neurons tested were sensitive to vanillin, of which 66.7% were also sensitive for cap, 25% for AITC, and 75% for men (fig. 2B).

Together, these findings indicate that TRPV1, TRPM8, and TRPA1 are expressed in different subsets of odorant-sensitive TG neurons. Consequently, these channels are likely to contribute to the observed odorant-induced activation of the neurons. The fact that neurons insensitive to TRP agonists nevertheless responded to odor stimulation indicates that further receptors are modulated by these compounds.

### Capsazepine and BCTC reduce the odorant-evoked depolarization of menthol- and capsaicin-sensitive TG neurons

**Figure 3:**
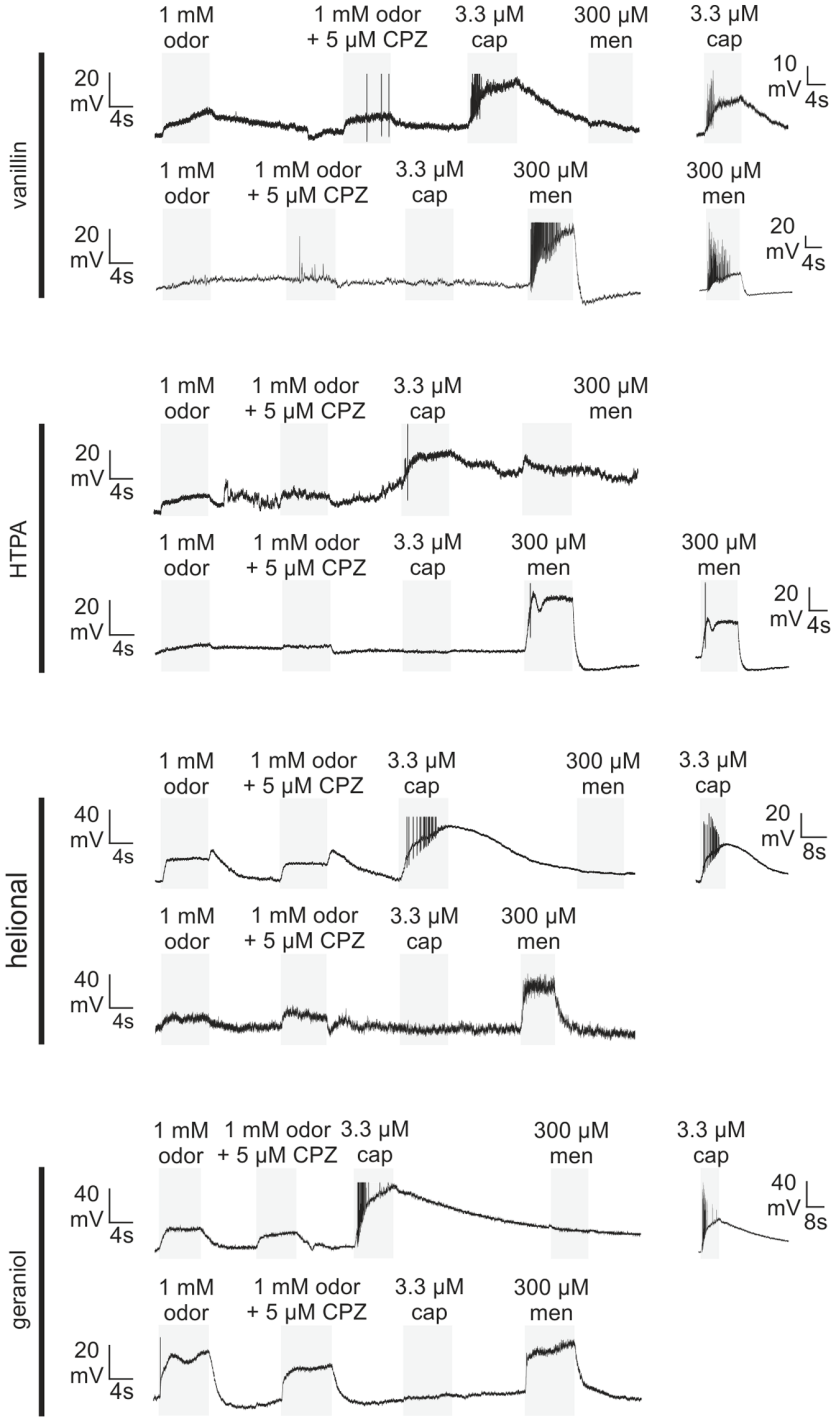
CC recording of cultured TG neurons stimulated with odorants in the presence and absence of CPZ. Exemplary CC recordings from cultured TG neurons upon stimulation with vanillin, HTPA, helional, and geraniol alone and with CPZ. Membrane potential changes and RMP of recorded neurons upon stimulation with: vanillin_cap-sensitive_: RMP: −45 mV; vanillin: Δ10 mV, vanillin+CPZ: Δ7 mV, cap: Δ20 mV, men: Δ0 mV; vanillin_men-sensitive_: RMP: −62 mV; vanillin: Δ0 mV, vanillin+CPZ: Δ0 mV, cap: Δ0 mV, men: Δ29,5 mV; HTPA_cap-sensitive_; RMP: −67 mV; HTPA: Δ14.6 mV, HTPA+CPZ: Δ10.3 mV, cap: Δ27.5 mV, Δ2.6 mV; HTPA_men-sensitive_; RMP: −61 mV; HTPA: Δ6.3 mV, Δ3.7 mV, cap: 0 mV, men: Δ31.4 mV; helional_cap-sensitive_: RMP: −73 mV; helional: Δ29.1 mV, helional+CPZ: Δ22 mV, cap Δ67 mV, men: 0 mV; helional_men-sensitive_: RMP: −50 mV; helional Δ7.2 mV, helional+CPZ: Δ7.8 mV, cap: Δ0 mV; men: Δ25 mV; geraniol_cap-sensitive_: RMP: −67 mV; geraniol: Δ18 mV, geraniol+CPZ: Δ14 mV, cap: Δ53 mV, men Δ0 mV; geraniol_men-sensitive_: RMP: −60 mV; geraniol: Δ17.6 mV, geraniol+CPZ: Δ14.5 mV, cap: Δ2 mV; men: Δ23 mV. Capsaicin- and menthol-evoked action potentials were cut at −30 mV (vanillin), −20 mV (HTPA), 0 mV (helional), and at −10 mV (geraniol). Stimulus applications are indicated by highlighted (*gray*) regions.

**Figure 4:**
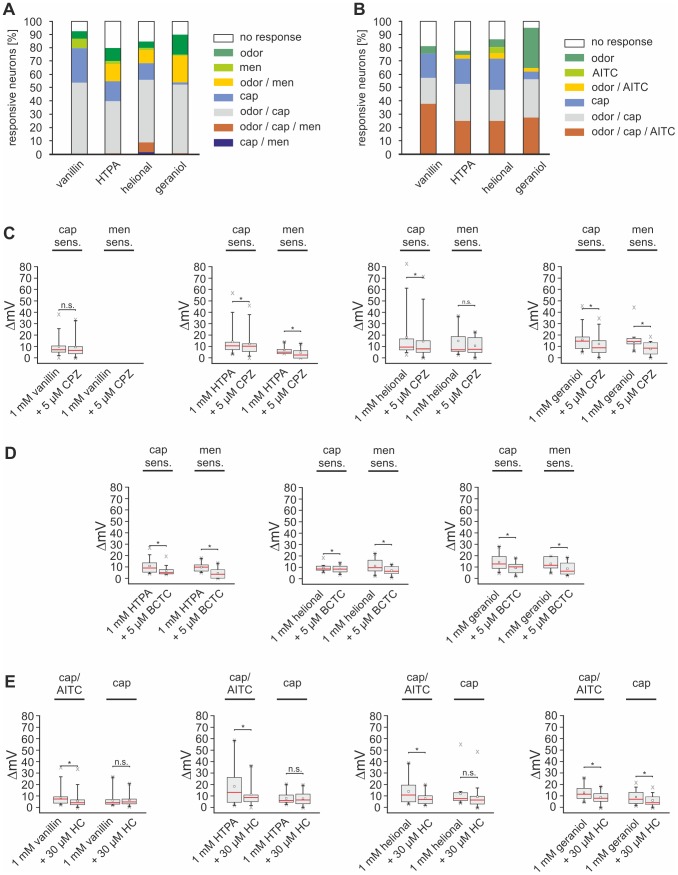
CPZ and HC inhibit odorant-evoked activations of cultured TG neurons. **A:** Bar chart, depicting the responsiveness (%) of TG neurons to the odorants vanillin (1 mM; n = 54), HTPA (1 mM; n = 125), helional (1 mM; n = 121), and geraniol (1 mM; n = 101), as well as to cap (3.3 µM) and men (300 µM). Detailed percentages can be derived from table S1. **B:** Bar chart, depicting the responsiveness (%) of TG neurons to the odorants vanillin (1 mM; n = 87), HTPA (1 mM; n = 68), helional (1 mM; n = 68), and geraniol (1 mM; n = 69) as well as to cap (3.3 µM) and AITC (50 µM). Detailed percentages can be derived from table S1. **(C-E)** Box plot diagrams depicting the odorant-evoked membrane potential depolarization in cultured TG neurons during whole-cell CC recordings in the presence and the absence of CPZ (C), BCTC (D) or HC (E). **C:** Recordings from menthol- and capsaicin-sensitive neurons were subdivided. Depolarizations triggered by vanillin (n = 54), HTPA (n = 70), helional (n = 76), and geraniol (n = 59) in the presence and in the absence of CPZ, as well as percentage values of inhibitions can be derived from table 4. **D:** Recordings from menthol- and capsaicin-sensitive neurons were subdivided. Depolarizations triggered by HTPA (n = 55), helional (n = 45), and geraniol (n = 42) in the presence and in the absence of BCTC as well as percentage values of inhibitions can be derived from table 4. **E:** Recordings from AITC/cap- and cap-sensitive neurons were subdivided, respectively (original traces are depicted in fig. S2). Depolarizations triggered by vanillin (n = 87), HTPA (n = 68), helional (n = 68), and geraniol (n = 69) in the presence and in the absence of HC can be derived from table 4.

Thus far, our data indicate that the TRP channels TRPV1, TRPM8, and TRPA1 are among the potential targets of the odorants tested. We used the TRPV1/TRPM8 antagonist capsazepine (CPZ, 5 µM) [11], [24], [49] to investigate if this substance inhibits odorant-evoked membrane potential changes in TG neurons during CC recordings. Functional expression of TRPV1 and/or TRPM8 in the cells was tested by challenging the neurons with cap (3.3 µM) and men (300 µM). Odorants, followed by odorants plus CPZ, cap, and finally men were each administered for 8 s (≥14 s interval) (fig. 3). Each neuron was stimulated with only one odorant. The percentages of responsive neurons are depicted in fig. 4A and table S1, amplitudes and number of repeated experiments can be derived from table 4. A potential desensitization of the odorant-evoked membrane potential depolarization was excluded by repetitive (3−4 times) administration of the same odorant (vanillin, HTPA, helional, geraniol) (data not shown).

**Table 4:** Amplitudes of TG neuron depolarizations triggered by administration of odorants and odorants plus the TRP channel antagonists CPZ, BCTC, and AITC.

	**capsaicin-sensitive neurons**			**menthol-sensitive neurons**		
	**depolarization by odorant [mV]**	**depolarization by odorant + CPZ [mV]**	**inhibition by CPZ [%]**	**depolarization by odorant [mV]**	**depolarization by odorant + CPZ [mV]**	**inhibition by CPZ [%]**
**vanillin**	7 +7/−5	6.5 +9.5/−3.9	16.1 +44.83/−0	no response	no response	no response
**HTPA**	10.6 +13.5/−7.5	10.2 +12/−6	8.16 +21.33/−0	5.8 +4.38/−7.7	3.25 +7.45/−0	62.12 +100/−22.22
**helional**	9.4 +16.3/−7.1	7.8 +14.5/−5	17.5 +34.62/−0	8.85 +18.2/−6.25	7.7 +17.45/−5.4	10.33 +34.91/−2.56
**geraniol**	14.75 +18.25/−8.4	8.7 +15.375/−5.2	23.55 +38.91/−12.14	15.1 +16.85/−12.68	8.4 +13.25/−4.48	33.65 +64.34/−21.07
	**capsaicin-sensitive neurons**			**menthol-sensitive neurons**		
	**depolarization by odorant [mV]**	**depolarization by odorant + BCTC [mV]**	**inhibition by BCTC [%]**	**depolarization by odorant [mV]**	**depolarization by odorant + BCTC [mV]**	**inhibition by BCTC [%]**
**vanillin**	not tested	not tested	not tested	not tested	not tested	not tested
**HTPA**	8.9 +13.2/−5.5	4.9 +7.4/−3.9	35.3 +41.6/−27.3	9.6 +11/−6.6	3.5 +7.4/−0	54.8 +100/−23.3
**helioal**	9.1 +11.6/−7.8	8 +11/−5.6	18.7 +32.4/−3.6	12.8 +17/−9.8	8.8 +12.2/−6.9	24 +32/−18.8
**geraniol**	12.65 +18.3/−8.9	10.2 +11.8/−5.5	36.9 +49.8/−22	11.6 +19/−9.2	6.3 +11.4/−4.2	48.3 +52/−29.3
	**capsaicin- and AITC-sensitive neurons**			**capsaicin-sensitive neurons**		
	**depolarization by odorant [mV]**	**depolarization by odorant + HC [mV]**	**inhibition by HC [%]**	**depolarization by odorant [mV]**	**depolarization by odorant + HC [mV]**	**inhibition by HC [%]**
**vanillin**	7.5 +9.38/−3.9	4.3 +6.7/−4.3 mV	23.12 +32.07/−11.73	4.3 +6.43/−3.11	2.6 +4.5/−0.9	not statistically significant
**HTPA**	13.41 +26.02/−5.06	9 +11.17/−5.54 mV	21.63 +45.02/−4.76	6.4 +10.25/−5.29	6.9 +11.73/−3.7	not statistically significant
**helional**	10,79 +18.46/−5.1	7.08 +9.48/−3.58	24.29 +19.65/−50.21	7.45 +18.46/−5.1	6.67 +9.48/−3.58	not statistically significant
**geraniol**	11.84 +16.46/−8.55	8.5 +11.89/−6.45	28.25 +42.39/−11.68	7.75 +13.7/−4.78	4.75 +9.73/−3.58	19.76 +43.97/−11.45

Vanillin-evoked depolarization was exclusively observed in cap- but not in men-sensitive neurons (fig. 4A and C, table 4 and S1). There was a tendency for lower responses in the presence of CPZ (p = 0.23; fig. 4C, table 4). This indicates that TRPV1 but not TRPM8 partially mediates the responses to vanillin, although further channels seem to be involved. HTPA caused activations in cap- and men-sensitive, but also in a further group of neurons that was cap- and men-insensitive (fig. 4A, table S1). CPZ reduced the HTPA-, helional-, and geraniol-evoked depolarization in cap- and in men-sensitive cells (p_HTPA/cap_ = 0.008, p_HTPA/men_ = 1.14*10^−7^; p_helional/cap_ = 9.76*10^−6^, p_helional/men_ = 0.13; p_geraniol/cap_ = 4.5*10^−5^, p_geraniol/men_ = 9.77*10^−4^; fig. 4C, table 4).

To further investigate a possible involvement of TRPM8 in the odorant-induced response, we exchanged CPZ by BCTC which is more commonly known as an antagonist for TRPM8 and investigated its effect on the depolarization triggered by the odorants. Since vanillin failed to activate menthol-sensitive neurons during CC recordings, we focused on investigating its effect on activations induced by HTPA, helional, and geraniol. All three substances triggered a depolarization in capsaicin- as well as menthol-sensitive neurons that were significantly smaller in the presence of BCTC (p_HTPA/cap_ = 4.88*10^−4^, p_HTPA/men_ = 7.81*10^−3^; p_helional/cap_ = 0.023, p_helional/men_ = 0.023; p_geraniol/cap_ = 7.81*10^−3^, p_geraniol/men_ = 9.77*10^−3^; fig. 4D, table 4).

These results indicate that both TRPV1 and TRPM8 are involved in the HTPA-, helional-, and geraniol-induced depolarization of TG neurons, whereas vanillin does not affect TRPM8. The low percentage of men/cap-responsive neurons observed in our recordings is in accordance with several previous studies [12], [14], [41], [50]. Nevertheless, several other groups described an overlapping sensitivity of TG neurons for menthol and capsaicin [51]–[53]

### HC-030031 reduces the odorant-induced depolarization in AITC- and capsaicin-sensitive TG neurons

The inhibition of the odorant-evoked depolarization in TG neurons by CPZ provided strong evidence for an involvement of TRPV1 and TRPM8. Our previous findings also revealed an overlap of responses to odorants and to AITC, indicating the additional activation of TRPA1.

To test for a role of TRPA1 in the odorant responses, we co-administered vanillin, HTPA, helional, and geraniol with the specific TRPA1 antagonist HC-030031 (HC, 30 µM) [54] in CC recordings of TG neurons (described above). At the end of every recording, cells were challenged by saturating concentrations of cap (3.3 µM) and AITC (50 µM), to test for the functional expression of TRPV1 and TRPA1 (fig. S2). Percentages of responsive neurons are depicted in fig. 4B and are listed in table S2. Response amplitudes and percentage inhibitions are listed in table 4. Most neurons depolarized by odorants were also sensitive for cap or both, cap and AITC (cap/AITC). Only geraniol triggered mentionable responses in a subgroup of AITC- and cap-insensitive neurons (n =  69; fig. 4E, table 4). HC reduced the amplitudes of vanillin-evoked responses in a subgroup of cells sensitive to cap- and AITC (p_cap/AITC_ = 0.0075; p_cap_ = 0.055; fig. 4E, table 4). Similar results were observed for HTPA and helional (HTPA: p_cap/AITC_ = 0.008, p_cap_ = 0.554; helional: p_cap/AITC_ = 4.38*10^−4­^, p_cap_ = 0.08 fig. 4E, table 4).

HC reduced the geraniol-evoked responses in cap- and AITC-sensitive neurons and interestingly also in a cap-sensitive population (p_cap/AITC_ = 2.9*10^−4^, p_cap_ = 6.2*10^−4^; fig. 4E, table 4).

Together with the results of recordings in which we co-administered CPZ, these findings indicate a direct contribution of TRPV1, TRPM8, and TRPA1 to the odorant-evoked depolarization of TG neurons. CPZ and HC failed to completely inhibit responses to odorants, which was also the case upon simultaneous administration of both antagonists (data not shown). According to these results, we suspect that in TG neurons further targets are modulated by the odorants tested, which is in accordance with our previously described Ca^2+^ imaging data.

### Odorants directly activate rTRPV1, rTRPM8, and TRPA1 in CHO cells

Next, we pharmacologically characterized effects of vanillin, HTPA, helional, and geraniol on rat TRP channels functionally expressed in CHO cells using whole-cell VC recordings in order to investigate the biophysical properties of the odorant-induced activations of TRPV1, TRPM8, and TRPA1. Since all three channels are desensitized by Ca^2+^ [55], the experiments were performed under Ca^2+^-free conditions. Odorant-induced currents were normalized to those evoked by saturating concentrations of cap (3.3 µM) for recordings of rTRPV1, men (300 µM) for recordings of TRPM8, or AITC (50 µM) for recordings of TRPA1. In the case of TRPV1 and TRPM8 all four substances were administered to the same cell in a fixed order (vanillin, HTPA, helional, geraniol). No effects were observed upon odorant-stimulation of CHO-cells solely expressing GFP (n = 17; data not shown).

**Figure 5:**
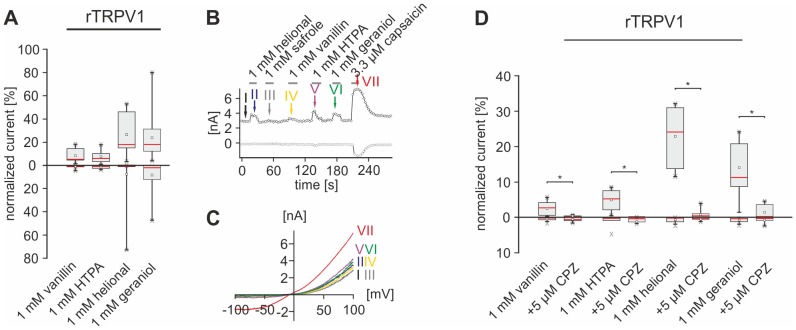
Odorants activate functionally expressed rTRPV1. **A:** Box plot diagram depicting normalized rTRPV1-mediated currents evoked by vanillin (n = 10), HTPA (n = 17), helional (n = 17), and geraniol (n = 17) during VC recordings. Outward currents recorded at +100 mV are depicted as upward facing bars, inward currents recorded at −100 mV are depicted as downward facing bars. Detailed values can be derived from table 5. **B:** Whole-cell VC recordings of CHO cells functionally expressing rTRPV1, challenged with vanillin, HTPA, helional, geraniol and cap. Amplitudes of currents at +100 (*black*) and −100 mV (*gray*) are plotted vs. time. Colored arrows and roman numerals assign to individual voltage ramps shown as current traces in the IV-plots depicted in **C**. Stimulus applications are indicated by gray bars. **D:** Box plot diagram depicting normalized rTRPV1-mediated currents triggered by vanillin (n = 11), HTPA (n = 9), helional (n = 10), and geraniol (n = 10) that are inhibited by CPZ (exemplary recordings are depicted in fig. S3A). Outward currents recorded at +100 mV are depicted as upward facing bars, inward currents recorded at −100 mV are depicted as downward facing bars. Detailed values can be derived from table 6.

All odorant-induced rTRPV1-mediated currents revealed a strong outward rectification with an E_rev_ ranging from −4.3 to −1.25 mV (fig. 5A-C) and occurred mainly in outward direction at +100 mV. The largest outward currents were induced by helional and geraniol, while those induced by vanillin and HTPA were visibly smaller (fig. 5A-C, table 5). Co-administration of CPZ nearly completely inhibited all odorant-evoked outward currents (p_vanillin_ = 9.8*10^−3^, p_HTPA_ = 3.9*10^−3^, p_helional_ = 1.95*10^−3^, p_geraniol_ = 1.95*10^−3^). Original traces of these recordings can be viewed in figure S3A. No statistically significant changes of inward currents were induced by CPZ (fig. 5D and S3A, table 6).

**Table 5:** Amplitudes of odorant-evoked in- (−100 mV) and outward (+100 mV) currents via functionally expressed rat TRPV1, TRPM8, and TRPA1.

	rTRPV1		rTRPM8		rTRPA1	
	I_norm_ at +100 mV [%]	I_norm_ at -100 mV [%]	I_norm_ at +100 mV [%]	I_norm_ at -100 mV [%]	I_norm_ at +100 mV [%]	I_norm_ at -100 mV [%]
**vanillin**	6.27 +12.77/−4.51	0 +0.46/−0	0.12 +0.93/−0	0 +0/−0	30.27 +51.04/−17.44	34.45 +66.78/−23.23
**HTPA**	6.59 +9.56/−3.48	1.05 +2.34/−1.05	5.89 +9.59/−3.9	0.08 +0.29/−0	75.95 +105.07/−70.1	19.99 +25.29/−9.36
**helional**	13.09 +18.02/−5.66	0 +0.66/−0	5.88 +5.84/−5.84	0.08 +0.29/−0	66.93 +100/−42.95	18.70 +21.19/−12.96
**geraniol**	19.11 +29.15/−12.83	2.1 +10.11/−0.12	29.02 +35.37/−25.63	1.28 +2.54/−0.57	12.03 +14.33/−7.6	1.09 +13.26/−0.25

**Table 6:** Values of odorant-evoked currents evoked by administration of odorants alone and odorants plus the TRP channel antagonists CPZ, BCTC, and AITC.

	rTRPV1				rTRPM8			
	I_norm_ at +100 mV [%]		I_norm_ at −100 mV [%]		I_norm_ at +100 mV [%]		I_norm_ at −100 mV [%]	
	odorant	odorant + CPZ	odorant	odorant + CPZ	odorant	odorant + CPZ	odorant	odorant + CPZ
**vanillin**	2.64 +4.04/−0.8	0.02 +0.33/−0	0.06 +0.66/−0	0.67 +0.85/−0.18	no response	no response	no response	no response
**HTPA**	5.23 +7.54/−1.82	0 +0/−0	0.66 +1.15/−0.57	0.63 +1.29/−0	3.82 +6.27/−2.31	0 +0.06/−0	2.03 +3.26/−0	0 +2.77/−0
**helional**	24.65 +30.51/−14.24	0 +0.31/−0	0.09 +0.31/−0	0 +0/−0	4.41 +6.94/−3.24	1.86 +2.66/−0.97	0.13 +0.87/−0	0 +0.12/−0
**geraniol**	13.1 +20.52/−9.49	0 +0/−0	0.08 +0.34/−0	0 +0/−0	18.52 +19.89/−9.74	7.42 +12,29/−3.61	0.3 +1.44/−0	0.15 +0.77/−0

	rTRPM8				rTRPA1			
	I_norm_ at +100 mV [%]		I_norm_ at −100 mV [%]		I_norm_ at +100 mV [%]		I_norm_ at −100 mV [%]	
	odorant	odorant + CPZ	odorant	odorant + CPZ	odorant	odorant + CPZ	odorant	odorant + CPZ
**vanillin**	no response	no response	no response	no response	23.9 +34.2/−15.3	0 +2.6/−0	43.7 +55.2/−30.1	0.1 +6.6/−0
**HTPA**	1.9 +5.1/−1.6	0 +0/−0	0.6 +3.9/−0	0 +0/−0	31 +53/−20.9	0 +4.9/−0	19.9 +40.7/−13.2	0 +7.5/−0
**helional**	6.3 +9.4/−3.3	0 +0/−0	1.5 +9/−0.1	0 +0/−0	44.4 +64.3/−40.2	18.8 +31.6/−11.1	20.4 +26.9/−9.3	7.1 +9.7/−1.9
**geraniol**	19.9 +24.2/−16.4	0 +0.4/−0	1.6 +2.5/−0.1	0 +0/−0	no response	no response	no response	no response

**Figure 6:**
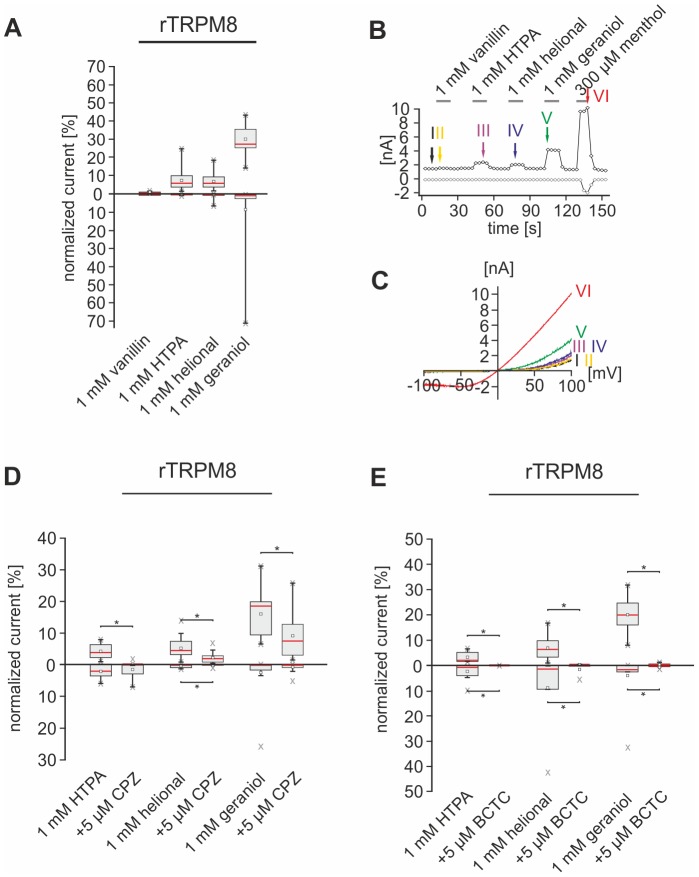
Odorants activate functionally expressed rTRPM8. **A:** Box plot diagram depicting normalized rTRPM8-mediated currents evoked by vanillin (n = 14), HTPA (n = 17), helional (n = 17), and geraniol (n = 17) during VC recordings. Outward currents recorded at +100 mV are depicted as upward facing bars, inward currents recorded at −100 mV are depicted as downward facing bars. Detailed values can be derived from table 5. **B:** Whole-cell VC recordings of CHO cells functionally expressing rTRPM8, challenged with vanillin, HTPA, helional, geraniol and men. Amplitudes of currents at +100 (*black*) and -100 mV (*gray*) are plotted vs. time. Colored arrows and roman numerals assign to individual voltage ramps shown as current traces in the IV-plots depicted in **C**. Stimulus applications are indicated by gray bars. **D:** Box plot diagram depicting normalized rTRPM8-mediated currents triggered by HTPA (n =  12), helional (n = 14), and geraniol (n = 14) that are inhibited by CPZ (exemplary recordings are depicted in fig. S4A). Outward currents recorded at +100 mV are depicted as upward facing bars, inward currents recorded at −100 mV are depicted as downward facing bars. Detailed values can be derived from table 6. **E**: Box plot diagram depicting normalized rTRPM8-mediated currents triggered by HTPA helional, and geraniol that are inhibited by BCTC (exemplary recordings are depicted in fig. S4B). Outward currents recorded at +100 mV are depicted as upward facing bars, inward currents recorded at −100 mV are depicted as downward facing bars. Detailed values can be derived from table 6.

Administration of vanillin failed to activate currents in CHO cells heterologously expressing rTRPM8. HTPA, helional, and geraniol mainly evoked outward-currents with marked outward-rectifications and an E_rev_ ranging from 8.45 to -3.68 mV (fig. 6A-C). The largest currents were elicited by geraniol, whereas the amplitudes of currents evoked by HTPA and helional were visibly smaller (fig. 6A-C, table 5). CPZ caused a nearly complete inhibition of HTPA-evoked outward currents, whereas those evoked by helional and geraniol were only reduced by ∼50% (p_HTPA_ = 4.88*10^−3^, p_helional_ = 1.1*10^−3^, p_geraniol_ = 1.22*10^−4^). No statistically significant effect was observed on inward currents triggered by HTPA and geraniol, whereas helional-induced inward currents were significantly reduced by CPZ (p = 0.039; fig. 6D and S4A, table 6). A more prominent effect on TRPM8-mediated currents triggered by administration of HTPA, helional, and geraniol was observed upon co-administration of 5 µM BCTC which nearly completely inhibited odorant-evoked currents in inward as well as outward direction (outward: p_HTPA_ = 3.9*10^−3^, p_helional_ = 1.95*10^−3^, p_geraniol_ = 1.95*10^−3^; inward: p_HTPA_ = 0.023, p_helional_ = 0.016, p_geraniol_ = 0.023; fig. 6E and S4B; table 6).

**Figure 7:**
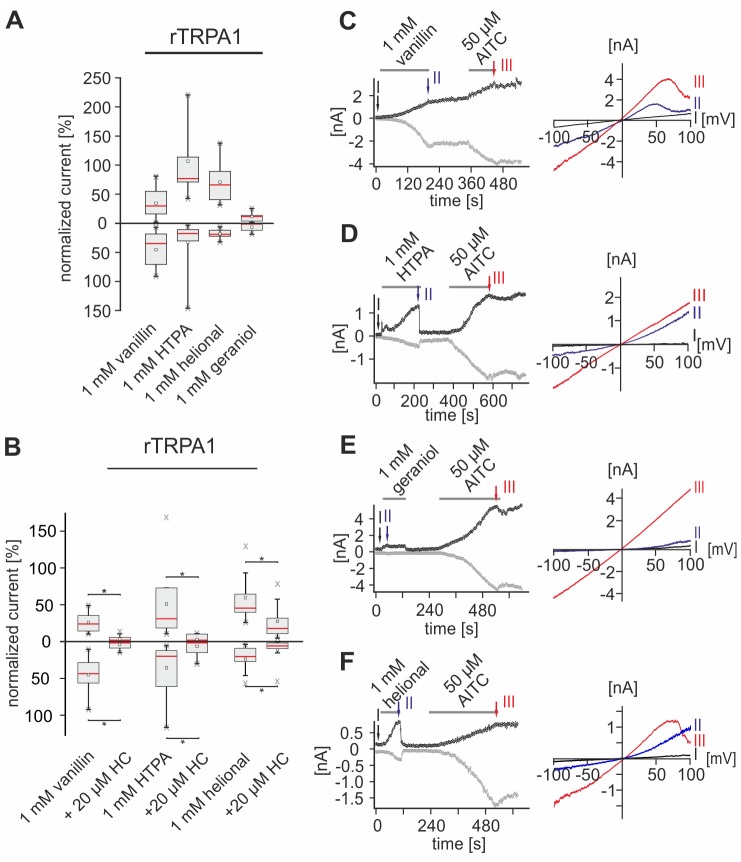
Odorants activate functionally expressed rTRPA1. **A:** Box plot diagram depicting normalized rTRPA1-mediated currents evoked by vanillin (n = 11), HTPA (n = 11), helional (n = 11), and geraniol (n = 8) during VC recordings. Outward currents recorded at +100 mV are depicted as upward facing bars, inward currents recorded at −100 mV are depicted as downward facing bars. Detailed values can be derived from table 5. **B:** Box plot diagram depicting normalized rTRPA1-mediated currents triggered by vanillin (n = 8), HTPA (n = 7), and helional (n = 9) that are inhibited by HC (exemplary recordings are depicted in fig. S3B). Outward currents recorded at +100 mV are depicted as upward facing bars, inward currents recorded at −100 mV are depicted as downward facing bars. Detailed values can be derived from table 6. **C-F:** Whole-cell VC recordings of CHO cells functionally expressing rTRPA1, challenged with vanillin (C), HTPA (D), helional (E), geraniol (F) and AITC. Amplitudes of currents at +100 (*black*) and −100 mV (*gray*) are plotted vs. time. Colored arrows and roman numerals assign to individual voltage ramps shown as current traces in the IV-plots depicted right sided, respectively. Stimulus applications are indicated by gray bars.

Apart from desensitization, Ca^2+^ is also involved in the gating of rTRPA1. Thus, rTRPA1-mediated currents reveal delayed current rise and decay times under Ca^2+^-free conditions [13], [23], [56]–[60]. Since this may prevent odorant-evoked currents from returning to baseline-level, we only applied one odorant followed by AITC in each recording (fig. 7C-F).

HTPA caused the strongest outward currents in rTRPA1-expressing cells, whereas vanillin evoked the largest inward currents. Helional induced currents mainly in outward direction. Geraniol-evoked currents were relatively small compared to those triggered by the other compounds (fig. 7A, C-F, table 5). E_rev_ of all currents ranged from 2.79 to −0.18 mV (fig. 7C-F). Co-administration of HC nearly completely inhibited vanillin- and HTPA-evoked outward and inward currents (out: p_vanillin_ = 0.0078, p_HTPA_ = 0.0156; in: p_vanillin_ = 0.0469, p_HTPA_ = 0.0391). The helional response was significantly reduced by ∼70% in the presence of HC (out: p = 0.039; in: p = 0.0391; fig. 7B and S3B, table 6). Since administration of geraniol induced only small currents, no further experiments were performed in order to investigate the effect of HC on these currents.

These results show that vanillin, HTPA, helional, and geraniol directly activate recombinantly expressed rTRPV1. rTRPM8 is directly activated by HTPA, helional, and geraniol, while rTRPA1 is activated by vanillin, HTPA, and helional. All odorant-induced currents mediated by these channels were significantly inhibited by specific antagonists.

Beyond that, we investigated the effect of vanillin, HTPA, helional and geraniol on human TRP-channels. Therefore, we performed the same VC recordings described above on CHO cells functionally expressing the human variants of the channels (fig. S5, table S2). No species-dependent differences were observed when comparing the amplitudes of TRPM8- and TRPA1-mediated currents (p-values can be derived from table S3). Despite minor differences seen in the response profiles of human and rat TRPV1, our findings provide evidence that the odorants we tested activate TRP channels from both species with similar selectivity.

### Inhibition of TASK1 and TASK3 by odorants

**Figure 8:**
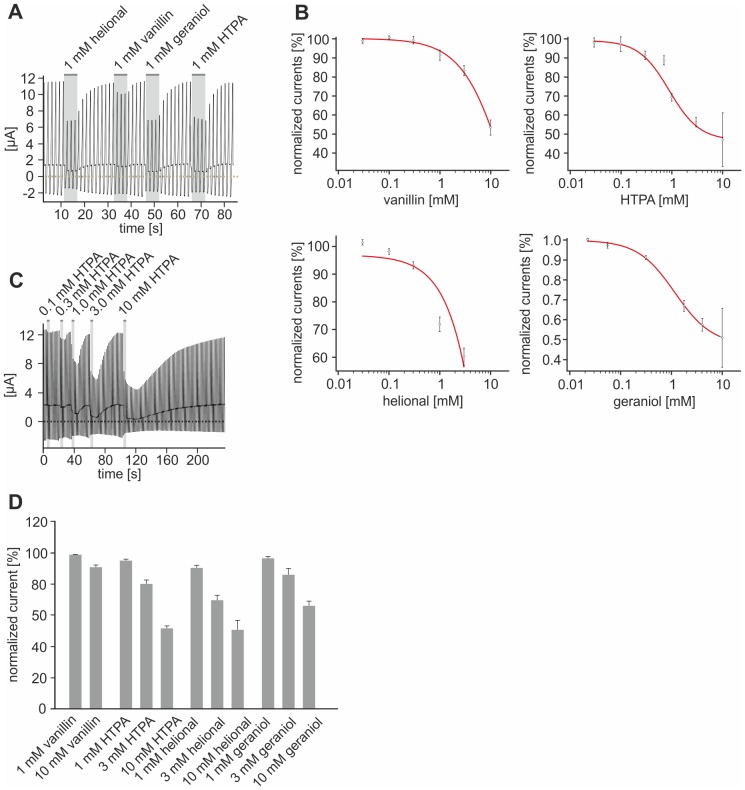
Odorants dose dependently inhibit TASK1 and TASK3. **A:** Exemplary VC recording of rTASK1-mediated voltage-driven currents upon administration of helional, vanillin, geraniol, and HTPA. **B:** Dose-response relations for vanillin, HTPA, helional, and geraniol, obtained from rTASK1 expressing *Xenopus laevis* oocytes during VC recordings. Compounds were administered in concentrations of 0.03, 0.1, 0.3, 1, 3, and 10 mM in ascending order. Data points present mean values and error bars present standard errors. **C:** Exemplary VC recording of TASK3-mediated voltage-driven currents during administration HTPA in arising concentrations. **D:** Bar diagram depicting the influence of varying concentrations of vanillin, HTPA, helional, and geraniol on voltage-driven currents via rTASK3 during VC recordings from *Xenopus laevis* oocytes. Bars present mean values and error bars present standard errors.

Since we were not able to completely inhibit odorant-induced membrane potential changes by TRP channel antagonists, we suspected a modulation of further target receptors and/or channels. Possible candidates are members of the two-pore-domain potassium (K_2_P, KCNK) channel family. Members of this family are highly expressed in sensory afferents [61] and some of them have been associated with typical trigeminal sensations, e.g. numbing and tingling [62]. Additionally, recent studies demonstrated that many stimuli that modulate TRP channels (e.g. pH, anandamide, hydroxy-α-sanshool, temperature, 2-APB) also modulate members of the K2P family [39], [63]–[66]. We therefore asked whether the rat K2P channels TASK1 (K2P3, KCNK3) and TASK3 (K2P9, KCNK9), whose mouse orthologues have been reported to be the target of the trigeminal agonist hydroxy-α-sanshool [62], could be affected by vanillin, HTPA, helional, and geraniol. Additionally, we tested the odorants javanol, sandranol and 2-Phenylethanol (PEE) that caused no effect on cultured trigeminal neurons. In order to address this question we performed two-electrode VC recordings on *Xenopus laevis* oocytes (n = 3-5) functionally expressing rTASK1 and rTASK3. As a first result, we found that voltage-induced responses of rTASK1 are inhibited by all four compounds tested (fig. 8A). Geraniol inhibited voltage-driven currents of rTASK1 with an IC_50_ of 0.58 ± 0.09 mM and an inhibition-threshold ranging between 0.03 and 0.1 mM. Comparable effects were seen for HTPA with an IC_50_ of 0.89 ± 0.12 mM and an inhibition threshold between 0.1 and 0.3 mM (fig. 8B). Since 10 mM vanillin and 10 mM helional were not sufficient to cause saturating effects on voltage-driven currents, we were unable to determine IC_50_ values for these odorants. However, the obtained data revealed that both substances inhibited voltage-driven outward currents in a dose-dependent manner and an inhibition-threshold for vanillin between 0.3 and 1 mM and for helional between 0.1 and 0.3 mM (fig. 8B).

Comparable results were obtained from oocytes functionally expressing rTASK3 (n = 3−5). A representative recording in which oocytes were challenged with different concentrations of HTPA is depicted in figure 8C. With exception of vanillin that induced only a minor inhibition of voltage-driven outward currents, the substances tested inhibited rTASK3 in a dose-dependent manner. However, none of the concentrations used resulted in a complete block of the currents. Nevertheless, HTPA, helional, and geraniol inhibited voltage-driven TASK3-mediated currents in a dose-dependent manner (fig. 8D).

Finally, we tested if sandalore, sandranol, javanol, and PEE that caused no direct effect on cultured trigeminal neurons could affect the basal activity of rTASK1 or rTASK3. In accordance with our previous findings, 1 mM sandalore, 1 mM javanol, and 1 mM PEE did not affect basal activity of TASK1 and TASK3 (data not shown). Only 1 mM sandranol caused a slight inhibition of TASK1 but not TASK3 (data not shown) that was far less pronounced than inhibitions triggered by vanillin, HTPA, helional, or geraniol.

In addition to TRP channels, we identified two K_2_P-channels, namely TASK1 and TASK3, which are targeted by HTPA, helional, and geraniol, while vanillin targeted exclusively TASK1. Since these channels are highly expressed in TG neurons, their inhibition is likely to contribute to the odorant-induced depolarization of these cells and the discrimination between different neuronal populations.

## Discussion

In the present study, we demonstrated differential expression of TRP channels in subpopulations of cultured TG neurons, and analyzed the modulation of the membrane potential of neurons in these subgroups by the odorants PEE, sandalore, sandranol, javanol, vanillin, HTPA, helional, and geraniol. Administration of vanillin, HTPA, helional, and geraniol caused the concentration-dependent depolarization of TG neurons while the other tested compounds caused no measureable effects.

The observed neuronal activations at least partially involved TRPV1, TRPA1, and TRPM8 channels expressed on the neuronal sub-populations, as demonstrated by the application of specific TRP channel antagonists. In combination with recordings from functionally expressed channels, we identified vanillin, HTPA, and helional as novel modulators for TRPV1, HTPA and helional for TRPM8, and vanillin, HTPA, and helional for TRPA1. Additionally, we confirmed the previously described activation of TRPM8 and TRPV1 by geraniol [24] and the lack of an activating effect of geraniol in concentrations of 1 mM on TRPA1 [25]. We further observed the inhibition of helional- and geraniol-evoked currents by the TRPA1 antagonist HC in cap- but not AITC-sensitive neurons. This may be caused by a slight inhibition of TRPV1 by HC as indicated by other authors [67]. In contrast, depolarizations caused by vanillin and HTPA were exclusively inhibited in AITC-sensitive neurons. This may hint to a higher importance of TRPA1 for the detection of vanillin and HTPA. This hypothesis is further supported by the finding that vanillin and HTPA caused stronger activation of functionally expressed TRPA1, than helional and geraniol. Onto the contrary, functionally expressed TRPV1, was significantly stronger affected by helional and geraniol than by vanillin and HTPA.

Upon investigating odorant-induced activations of heterologously expressed TRPV1 and TRPM8 we observed mainly outward currents that were nearly completely inhibited by CPZ, and BCTC. In contrast, this was not always the case for currents in inward direction, especially for those mediated by TRPV1. TRPV1 as well as TRPM8 are activated by voltage [68]. Inward currents were recorded at a potential of −100 mV, while CC recordings were in average performed at more physiological neuronal resting membrane potentials of −59.1 −52.2/−66 mV. Due to this more positive potential, voltage-sensitive TRP channels expressed in neurons are likely to feature a higher activatability for odorants than channels expressed in CHO cells at a holding potential at −100 mV. Additionally, several other factors expressed in sensory neurons may contribute to a higher activatability of TRP channels expressed in sensory neurons. One possible modulator is the protein Pirt (phosphoinositide regulator of TRP) that was shown to sensitize TRPV1 as well as TRPM8 [69], [70].

As further channels potentially contributing to odorant-evoked membrane potential changes we identified the K_2_P channels TASK1 and TASK3. Both are often co-expressed with TRPV1 and TRPV1/TRPA1 [62], [71], [72]. TASK1 was dose-dependently inhibited by vanillin, HTPA, helional, and geraniol. With the exception of vanillin that caused no effect, similar results were obtained for TASK3. K_2_P channels stabilize the resting membrane potential of TG neurons below firing threshold and expedite repolarization of action potentials [73]. Permanently opened K^+^ channels provide a constant K^+^ efflux driven by the electrochemical gradient. Inhibition of this efflux causes the intracellular accumulation of positively charged K^+^, thereby depolarizing the membrane potential. Due to their co-expression and the finding that TRP and K_2_P channels are often modulated by similar modalities (e.g. pH, and noxious temperatures [66], [74]–[76]), both groups of channels may interact in a synergistic fashion.

Under Cs^+^-based conditions, we observed two distinct neuronal populations which responded to odorant stimulation either with an activation or an inhibition of currents. The inhibition was exclusively observed in VC recordings under these conditions and likely result from the inhibition of voltage-driven background currents for instance via other TRP channels that may be inhibited by the odorants used, possibly TRPM4 that was shown to be activated at positive potentials [77] and whose mRNA expression was verified in DRG sensory neurons [78].

Although odorants generally activate the olfactory system at concentrations well below the trigeminal thresholds [36], the general activation of trigeminal fibers is well documented. Anosmics lacking all fine odor discrimination skills may still maintain the ability to roughly distinguish between different odor categories [27], [28] and olfactory adaptation may occur when the olfactory system sensitizes rapidly in reaction to the prolonged presence of many odorants in persons with an intact olfactory system [79]. We did not observe similar sensitization in the trigeminal system. Thus, the capacity of the trigeminal system to detect odorants at high concentrations may be relevant in situations in which the olfactory system is permanently eliminated or temporarily ineffective due to sensitization. Furthermore, it was shown that high concentrations of several volatile compounds can damage the olfactory epithelium [80]. This damage may result in degeneration, keratinization, or acute and/or chronic inflammation of the epithelium and may finally result in olfactory dysfunction or even olfactory loss. This is another situation, in which activation of the trigeminal system by high odorant concentrations may be helpful. Thus, besides its function as an alerting system, the trigeminal systems ability to discriminate between different chemicals may become particular useful in specific situations.

While the alerting functions of the trigeminal system are well described, nearly nothing is known about the discriminative skills of the trigeminal system with respect to different compounds and their underlying detection mechanisms. A recent study used voltage-sensitive dyes to monitor stimulus-specific activity patterns that occurred in a stimulus specific manner within the rat TG [33]. While these patterns may illustrate the discriminative abilities of the trigeminal system, the underlying molecular mechanisms remain elusive.

In the current study, we show that different odorants modulate specific combinations of TRP and K2P channels. This differential modulation is likely to result in activation of distinct sub-populations of TG neurons which in turn may underlie the odorant-specific activity patterns in the TG previously observed. Further processing may transfer the induced activity patterns to higher brain regions, where they may be experienced consciously and unconsciously as sensations related to the specific volatiles. Our findings that individual antagonists cause significant but incomplete effects that were much smaller than those observed on recombinant channels suggest a high degree of promiscuity on molecular targets. This high overlap minimizes possible combinations of neurons activated by distinct odorants which is not surprising since the discriminative abilities of the trigeminal system are much weaker than those of for instance the olfactory system.

Since only minor differences where apparent between human and rat TRP channels, the discriminatory power observed in the rat TG by Rothermel et al. [33], may in a similar way be present in humans. Thus, these channels are likely to contribute to different activity patterns in the human TG, providing an explanation for the discriminative skills of anosmics [27].

Although vanillin has long been viewed as an exclusively olfactory stimulus [7], [34], [35], vanillin-evoked activity within the TG *in vivo* was recently shown [33]. Incomplete knowledge exists about the mechanisms underlying the detection of vanillin and other odorants by trigeminal afferents. Although it was shown that TRPV3 is activated by high vanillin-concentrations (>10 mM) [43], those used *in vivo* were considerably lower. Furthermore, TRPV3-expression in TG neurons is controversially discussed [25], [81], rendering it rather improbable that TRPV3 caused the observed trigeminal activity. Our study now identifies vanillin as a novel modulator for TRPV1 and TRPA1, and reveals that vanillin inhibits TASK1. Since these interactions require lower concentrations than the activation of TRPV3, modulation of these channels is more likely to cause the observed activity.

Distinct neuronal populations of trigeminal neurons can be distinguished based on their differential expression of TRP channels. In summary, our results show that these subgroups are specifically activated by different volatile compounds. The activation may be responsible for, or at least contribute to, the molecular basis underlying the previously observed odorant-specific patterns of neuronal activity within the TG. We show that the activation of TG neurons by odorants is significantly mediated by different TRP channels, and that inhibition of TASK1 and TASK3 by odorants might further depolarize these neurons. Our findings provide a relevant contribution to understand the chemosensory properties of TG neurons. They may also shed light on trigeminal system function in general and its medical relevance for indications such as migraine. The existence of a potential link to migraine is indicated by the observation that various volatiles trigger migraine, likely via TRPA1-dependent mechanisms [82].

## Supporting Information

Figure S1 **Dose dependent intracellular Ca^2+^ increases of cultured TG neurons.** Concentration-response curves for vanillin, HTPA, helional, and geraniol obtained during Ca^2+^ imaging experiments from Fura2/AM-loaded TG neurons in a randomly chosen field. Cells were challenged (10 s each concentration; interval  =  120 s) with increasing concentrations of vanillin and HTPA (0.01, 0.1, 1, 3, 10 mM) as well as helional and geraniol (0.001, 0.1, 1, 3, 10 mM).
(TIFF)

Figure S2 **CC recording of cultured TG neurons stimulated with odorants in the presence and absence of HC.** Exemplary CC recordings from cultured TG neurons upon administration of vanillin, HTPA, helional, and geraniol alone and with HC. Capsaicin-induced action potentials were cut at 10 mV (vanillin), −40 mV (HTPA), and at −20 mV (geraniol). Stimulus applications are indicated by highlighted (*gray*) regions. Membrane potential changes and RMP of recorded neurons upon stimulation with: vanillin: RMP −48 mV; vanillin: Δ12.24 mV, vanillin+HC: Δ8.7 mV; AITC: Δ5.5 mV; cap: Δ54.6 mV; HTPA: RMP: −62.8 mV; HTPA: Δ13.95 mV, HTPA+HC: Δ10.52 mV, AITC: Δ 6.12 mV, cap: Δ25.5 mV; helional: RMP: −54.8 mV; helional: Δ38.7 mV, helional+HC: Δ19.76 mV, AITC: 9 mV, cap: 28.1 mV; geraniol: −67.3 mV; geraniol: Δ4.1 mV, geraniol+HC: Δ3.9 mV, AITC: 2 mV, cap: Δ41.3 mV.
(TIFF)

Figure S3 **Odorant-evoked currents via rat TRPV1 and rat TRPA1 are inhibited by specific antagonists.** Exemplary whole-cell VC recordings performed on CHO cells heterologously expressing rTRPV1 (A), or rTRPA1 (B) challenged with vanillin, HTPA, helional, and geraniol, and one positive stimulus (rTRPV1: cap; rTRPA1: AITC) in the presence and the absence of specific antagonists (rTRPV1: CPZ; rTRPA1: HC). Amplitudes of currents at +100 (*black*) and −100 mV (*gray*) are plotted vs. time. Colored arrows and roman numerals assign individual voltage ramps shown as current traces in the IV-plots depicted underneath, respectively. Stimulus applications are indicated by gray bars.
(TIFF)

Figure S4 **Odorant-evoked currents via rat TRPM8 are inhibited by CPZ and BCTC.** Exemplary whole-cell VC recordings performed on CHO cells heterologously expressing rTRPM8 challenged with HTPA, helional, geraniol and menthol in the presence and the absence of the antagonists CPZ (A) or BCTC (B). Amplitudes of currents at +100 (*black*) and −100 mV (*gray*) are plotted vs. time. Colored arrows and roman numerals assign individual voltage ramps shown as current traces in the IV-plots depicted underneath, respectively. Stimulus applications are indicated by gray bars.
(TIFF)

Figure S5 **Odorants activate functionally expressed hTRPV1, hTRPM8, and hTRPA1. A,C,E:** Box plot diagrams depicting normalized hTRPV1- (A), hTRPM8- (C), and hTRPA1-mediated (E) currents evoked by vanillin, HTPA, helional, and geraniol during whole-cell VC recordings. Outward currents recorded at +100 mV are depicted by upward facing bars, inward currents recorded at −100 mV are depicted by downward facing bars. Detailed values can be derived from table S2. hTRPV1: n_vanillin_ =  20, n_HTPA_ = 20, n_helional_ = 20, n_geraniol_ = 20; hTRPM8: n_vanillin_ = 13, n_HTPA_ = 13, n_helional_ = 13, n_geraniol_ = 13; hTRPA1: n_vanillin_ = 5; n_HTPA_ = 6, n_helional_ = 7, n_geraniol_ = 6. **B,D,F:** Exemplary whole-cell VC recordings performed on CHO cells heterologously expressing hTRPV1 (B), hTRPM8 (D), or hTRPA1 (F) challenged with vanillin, HTPA, helional, geraniol, and one positive stimulus (hTRPV1: cap; hTRPM8: men; hTRPA1: AITC). Amplitudes of currents at +100 (*black*) and −100 mV (*gray*) are plotted vs. time. Colored arrows and roman numerals assign individual voltage ramps shown as current traces in the IV-plot depicted underneath, respectively. Stimulus applications are indicated by gray bars.
(TIFF)

Table S1 Responsiveness (%) of TG neurons to the odorants vanillin, HTPA, helional, and geraniol (1 mM each), as well as to cap and men or to cap and AITC.
(DOCX)

Table S2 Amplitudes of odorant-evoked in- (−100 mV) and outward (+100 mV) currents via functionally expressed human TRPV1, TRPM8, and TRPA1. In: inwardly directed currents at −100 mV, out: outwardly directed currents at +100 mV.
(DOCX)

Table S3 P-values depicting statistical comparisons (U-test) of odorant-induced currents recorded from functionally expressed rat and human TRP channels. In: inwardly directed currents at −100 mV, out: outwardly directed currents at +100 mV.
(DOCX)
