# Supplementary figures and images for: Transient Receptor Potential Channels Encode Volatile Chemicals Sensed by Rat Trigeminal Ganglion Neurons
Source: PLoS One. 2013 Oct 21;8(10):e77998. doi: 10.1371/journal.pone.0077998 (PMC3804614; doi:10.1371/journal.pone.0077998)

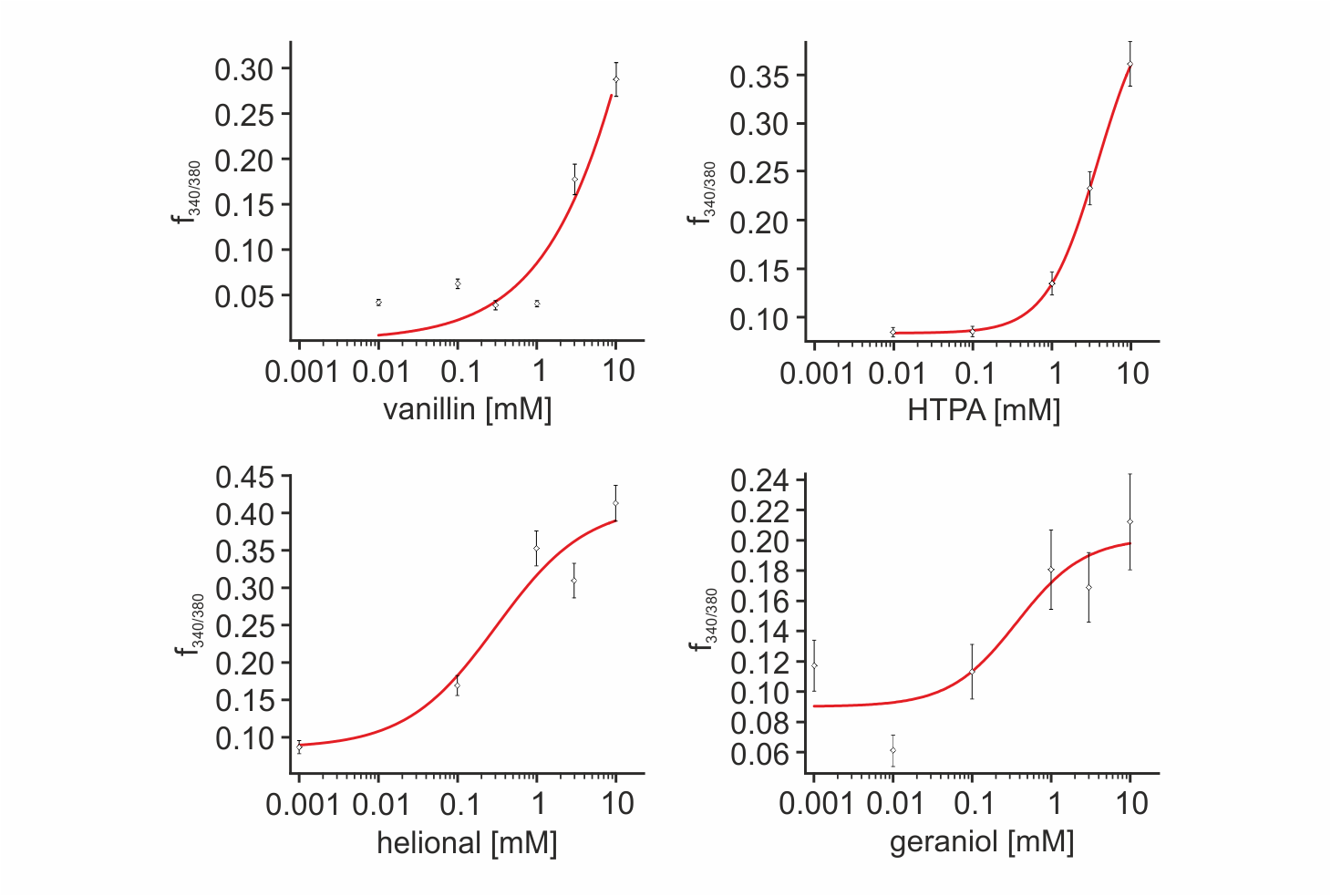

Supplement: Figure S1 — Dose dependent intracellular Ca2+ increases of cultured TG neurons. Concentration-response curves for vanillin, HTPA, helional, and geraniol obtained during Ca2+ imaging experiments from Fura2/AM-loaded TG neurons in a randomly chosen field. Cells were challenged (10 s each concentration; interval = 120 s) with increasing concentrations of vanillin and HTPA (0.01, 0.1, 1, 3, 10 mM) as well as helional and geraniol (0.001, 0.1, 1, 3, 10 mM). (TIFF) [file pone.0077998.s001.tiff]

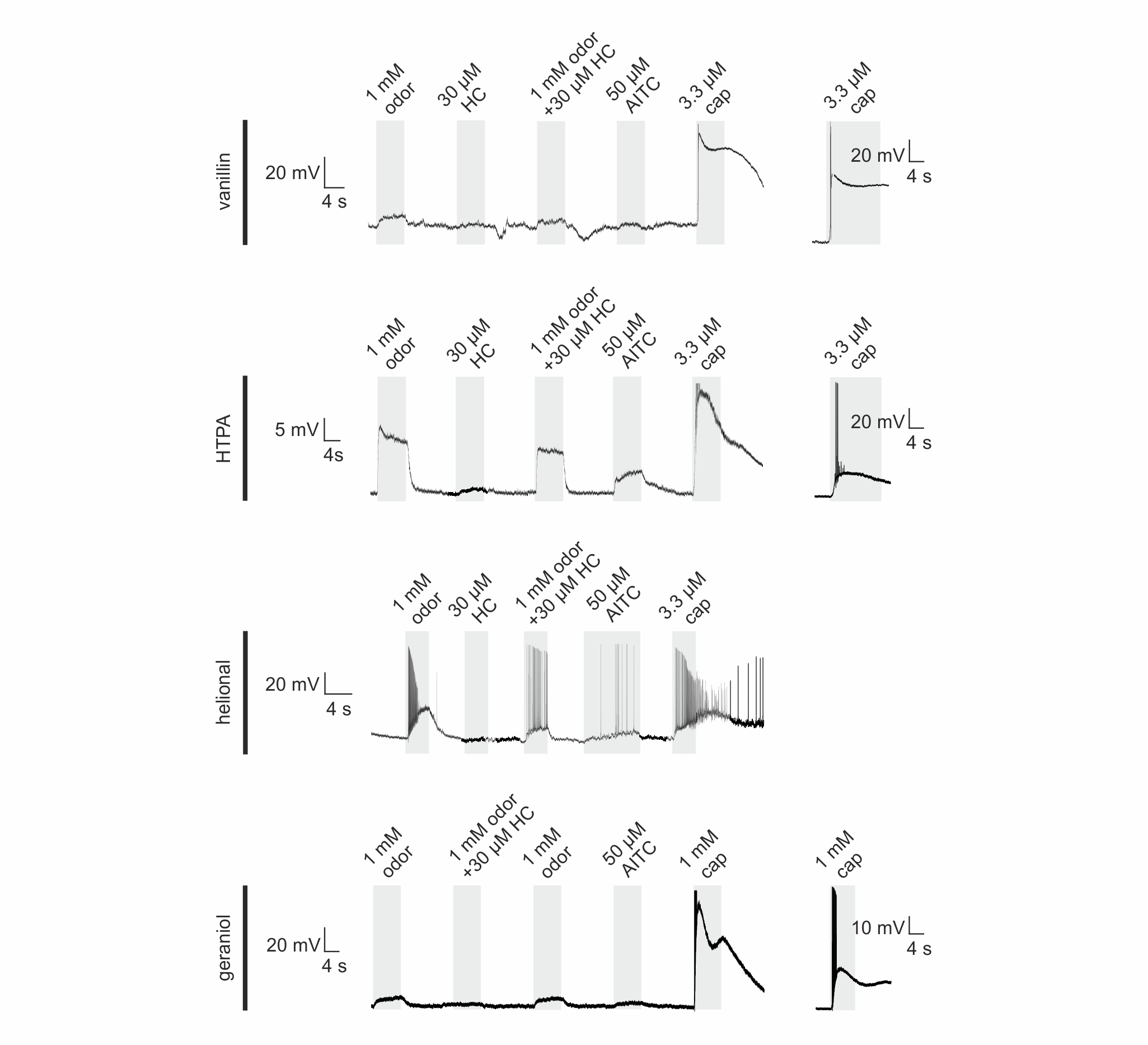

Supplement: Figure S2 — CC recording of cultured TG neurons stimulated with odorants in the presence and absence of HC. Exemplary CC recordings from cultured TG neurons upon administration of vanillin, HTPA, helional, and geraniol alone and with HC. Capsaicin-induced action potentials were cut at 10 mV (vanillin), −40 mV (HTPA), and at −20 mV (geraniol). Stimulus applications are indicated by highlighted (gray) regions. Membrane potential changes and RMP of recorded neurons upon stimulation with: vanillin: RMP −48 mV; vanillin: Δ12.24 mV, vanillin+HC: Δ8.7 mV; AITC: Δ5.5 mV; cap: Δ54.6 mV; HTPA: RMP: −62.8 mV; HTPA: Δ13.95 mV, HTPA+HC: Δ10.52 mV, AITC: Δ 6.12 mV, cap: Δ25.5 mV; helional: RMP: −54.8 mV; helional: Δ38.7 mV, helional+HC: Δ19.76 mV, AITC: 9 mV, cap: 28.1 mV; geraniol: −67.3 mV; geraniol: Δ4.1 mV, geraniol+HC: Δ3.9 mV, AITC: 2 mV, cap: Δ41.3 mV. (TIFF) [file pone.0077998.s002.tiff]

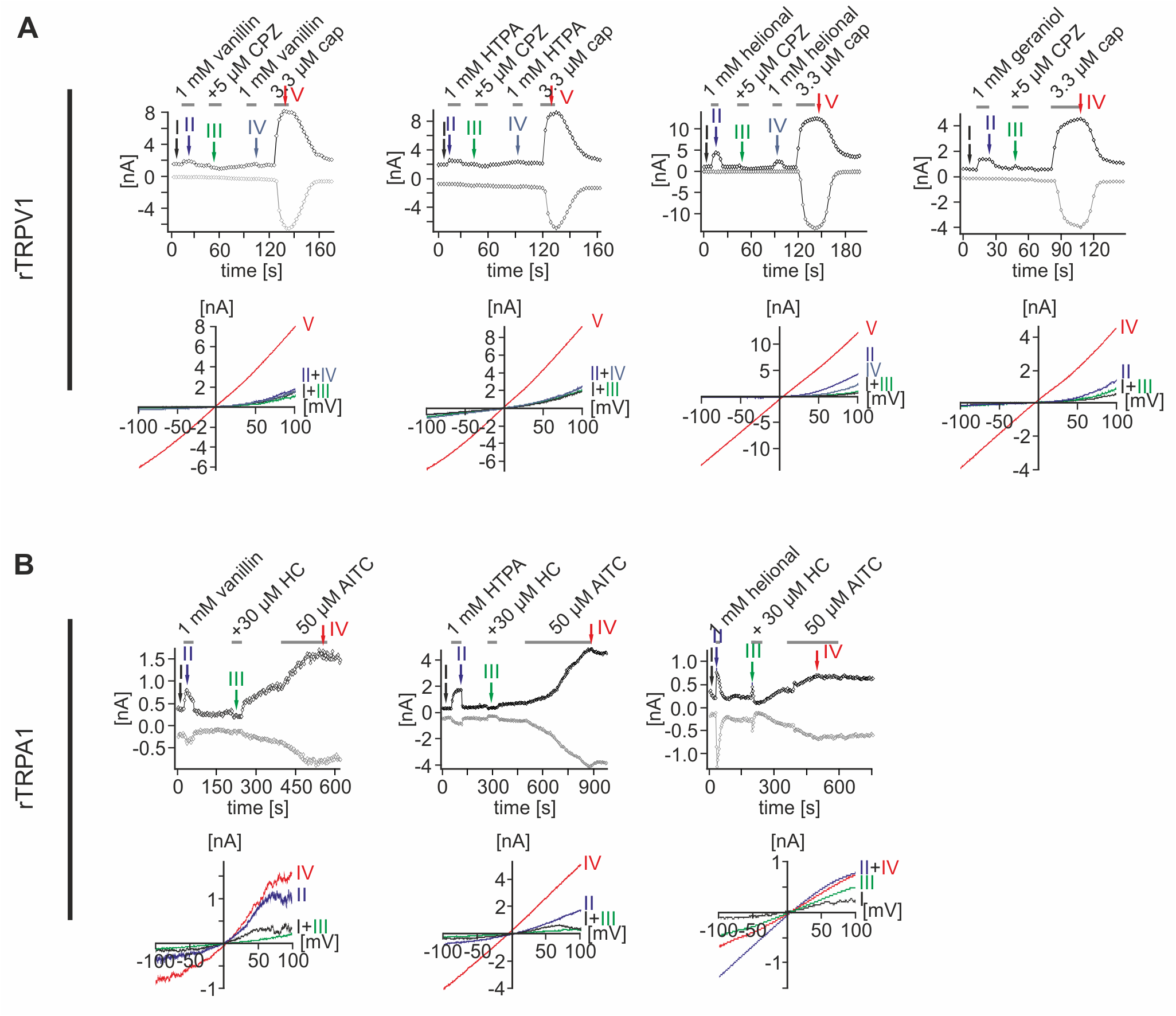

Supplement: Figure S3 — Odorant-evoked currents via rat TRPV1 and rat TRPA1 are inhibited by specific antagonists. Exemplary whole-cell VC recordings performed on CHO cells heterologously expressing rTRPV1 (A), or rTRPA1 (B) challenged with vanillin, HTPA, helional, and geraniol, and one positive stimulus (rTRPV1: cap; rTRPA1: AITC) in the presence and the absence of specific antagonists (rTRPV1: CPZ; rTRPA1: HC). Amplitudes of currents at +100 (black) and −100 mV (gray) are plotted vs. time. Colored arrows and roman numerals assign individual voltage ramps shown as current traces in the IV-plots depicted underneath, respectively. Stimulus applications are indicated by gray bars. (TIFF) [file pone.0077998.s003.tiff]

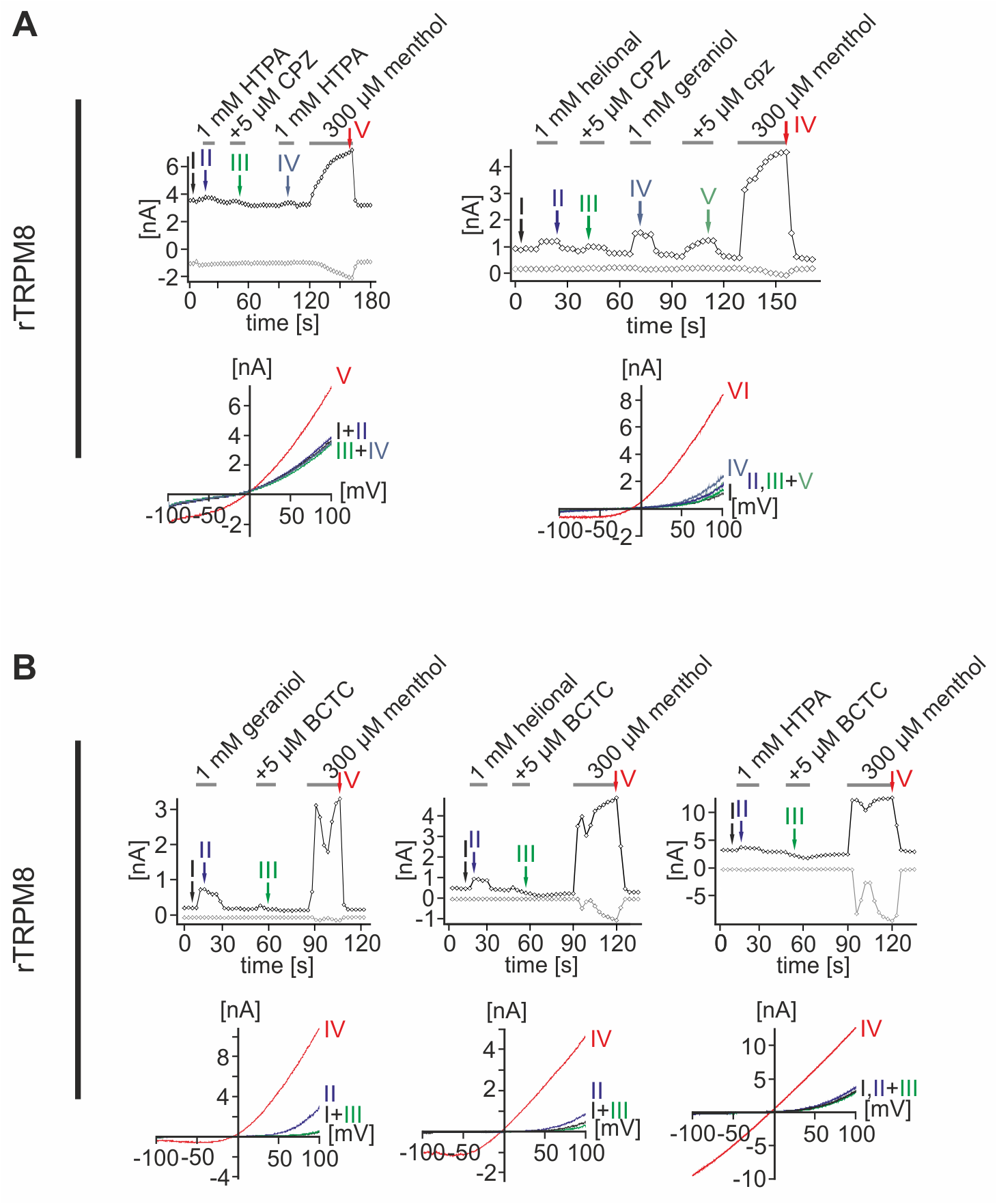

Supplement: Figure S4 — Odorant-evoked currents via rat TRPM8 are inhibited by CPZ and BCTC. Exemplary whole-cell VC recordings performed on CHO cells heterologously expressing rTRPM8 challenged with HTPA, helional, geraniol and menthol in the presence and the absence of the antagonists CPZ (A) or BCTC (B). Amplitudes of currents at +100 (black) and −100 mV (gray) are plotted vs. time. Colored arrows and roman numerals assign individual voltage ramps shown as current traces in the IV-plots depicted underneath, respectively. Stimulus applications are indicated by gray bars. (TIFF) [file pone.0077998.s004.tiff]

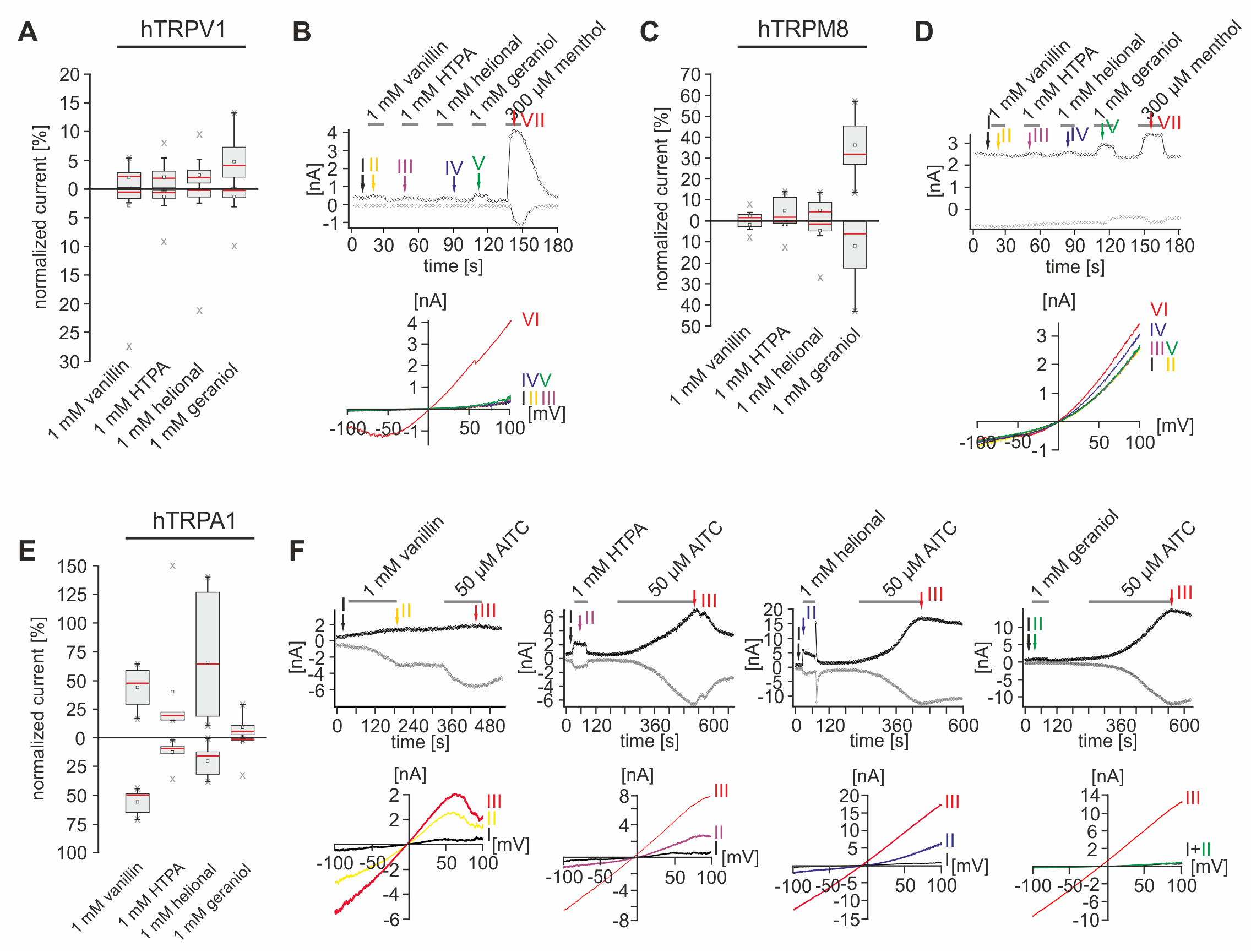

Supplement: Figure S5 — Odorants activate functionally expressed hTRPV1, hTRPM8, and hTRPA1. A,C,E: Box plot diagrams depicting normalized hTRPV1- (A), hTRPM8- (C), and hTRPA1-mediated (E) currents evoked by vanillin, HTPA, helional, and geraniol during whole-cell VC recordings. Outward currents recorded at +100 mV are depicted by upward facing bars, inward currents recorded at −100 mV are depicted by downward facing bars. Detailed values can be derived from table S2. hTRPV1: nvanillin = 20, nHTPA = 20, nhelional = 20, ngeraniol = 20; hTRPM8: nvanillin = 13, nHTPA = 13, nhelional = 13, ngeraniol = 13; hTRPA1: nvanillin = 5; nHTPA = 6, nhelional = 7, ngeraniol = 6. B,D,F: Exemplary whole-cell VC recordings performed on CHO cells heterologously expressing hTRPV1 (B), hTRPM8 (D), or hTRPA1 (F) challenged with vanillin, HTPA, helional, geraniol, and one positive stimulus (hTRPV1: cap; hTRPM8: men; hTRPA1: AITC). Amplitudes of currents at +100 (black) and −100 mV (gray) are plotted vs. time. Colored arrows and roman numerals assign individual voltage ramps shown as current traces in the IV-plot depicted underneath, respectively. Stimulus applications are indicated by gray bars. (TIFF) [file pone.0077998.s005.tiff]
