# Supplementary material for: Transient Receptor Potential Channels Encode Volatile Chemicals Sensed by Rat Trigeminal Ganglion Neurons
Source: PLoS One. 2013 Oct 21;8(10):e77998. doi: 10.1371/journal.pone.0077998 (PMC3804614; doi:10.1371/journal.pone.0077998)
Supplement: Table S1 — Responsiveness (%) of TG neurons to the odorants vanillin, HTPA, helional, and geraniol (1 mM each), as well as to cap and men or to cap and AITC. (DOCX) [file pone.0077998.s006.docx]

**Table S1:** Responsiveness (%) of TG neurons to the odorants vanillin, HTPA, helional, and geraniol (1 mM each), as well as to cap and men or to cap and AITC.

|  | **sensitivity for** | | | | | | | | |
| --- | --- | --- | --- | --- | --- | --- | --- | --- | --- |
|  | **odorant, men, and cap** | **odorant and cap** | | **cap only** | **odorant and men** | **men only** | **odorant only** | **cap and men** | **no response** |
| **vanillin (n=54)** | 0.0% | 53.7% | | 25.9% | 0.0% | 7.4% | 5.6% | 0.0% | 7.4% |
| **HTPA (n=125)** | 0.8% | 39.2% | | 15.2% | 12.8% | 2.4% | 9.6% | 0.0% | 20% |
| **helional (n=121)** | 7.4% | 47.1% | | 12.4% | 9.9% | 1,7% | 5% | 1.7% | 14.9% |
| **geraniol (n=101)** | 0.0% | 1% | | 2% | 19.8% | 1% | 14.9% | 1% | 9.9% |
|  | **sensitivity for** | | | | | | | | |
|  | **odorant, AITC, and cap** | | **odorant and cap** | **cap only** | **odorant and AITC** | **AITC only** | **odorant only** | **cap and AITC** | **no response** |
| **vanillin (n=87)** | 37.9% | | 19.5% | 18.4% | 0.0% | 0.0% | 5.8% | 0.0% | 18.4% |
| **HTPA (n=68)** | 25.0% | | 27.9% | 19.1% | 2.9% | 0.0% | 2.9% | 0.0% | 22.1% |
| **helional (n=68)** | 25.0% | | 23.5% | 23.5% | 4.4% | 4.4% | 5.9% | 0.0% | 13.2% |
| **geraniol (n=69)** | 27.5% | | 29.0% | 5.8% | 2.9% | 0.0% | 30.4% | 0.0% | 4.4% |
