# Supplementary material for: Transient Receptor Potential Channels Encode Volatile Chemicals Sensed by Rat Trigeminal Ganglion Neurons
Source: PLoS One. 2013 Oct 21;8(10):e77998. doi: 10.1371/journal.pone.0077998 (PMC3804614; doi:10.1371/journal.pone.0077998)
Supplement: Table S2 — Amplitudes of odorant-evoked in- (−100 mV) and outward (+100 mV) currents via functionally expressed human TRPV1, TRPM8, and TRPA1. In: inwardly directed currents at −100 mV, out: outwardly directed currents at +100 mV. (DOCX) [file pone.0077998.s007.docx]

**Table S2:** Amplitudes of odorant-evoked in- (-100 mV) and outward (+100 mV) currents via functionally expressed human TRPV1, TRPM8, and TRPA1. In: inwardly directed currents at -100 mV, out: outwardly directed currents at +100 mV.

|  | **hTRPV1** | | **hTRPM8** | | **hTRPA1** | |
| --- | --- | --- | --- | --- | --- | --- |
|  | **I_norm_ at +100 mV [%]** | **I_norm_ at -100 mV [%]** | **I_norm_ at +100 mV [%]** | **I_norm_ at -100 mV [%]** | **I_norm_ at +100 mV [%]** | **I_norm_ at -100 mV [%]** |
| **vanillin** | 2.63 +3.02/‑1.1 | 0.44 +1.93/‑0 | 1.62 +3.2/‑0 | 0 +2.68/‑0 | 54.33 +58.65/‑39.85 | 49.57 +64.57/‑48.95 |
| **HTPA** | 1.9 +3.05/‑0.39 | 0.64 +1.24/‑0.28 | 1.57 +11.14/‑018 | 0 +0.93/‑0 | 19.5 +12.54/‑16.44 | 10.38 +14.06/‑8.74 |
| **helional** | 1.94 +3.22/‑1 | 0.12 +1.34/‑0 | 4.34 +8.94/‑0.22 | 1.4 +4.76/‑0 | 64.35 +97.11/‑25.52 | 16.46 +32.01/‑13.9 |
| **geraniol** | 4.1 +7.15/‑2.18 | 0.27 +1.34/‑0 | 31.87 +45.36/‑26.96 | 6.07 +22.55/‑0 | 5.28 +8.79/‑2.95 | 0.12 +2.12/‑0.02 |
