# Supplementary material for: Transient Receptor Potential Channels Encode Volatile Chemicals Sensed by Rat Trigeminal Ganglion Neurons
Source: PLoS One. 2013 Oct 21;8(10):e77998. doi: 10.1371/journal.pone.0077998 (PMC3804614; doi:10.1371/journal.pone.0077998)
Supplement: Table S3 — P-values depicting statistical comparisons (U-test) of odorant-induced currents recorded from functionally expressed rat and human TRP channels. In: inwardly directed currents at −100 mV, out: outwardly directed currents at +100 mV. (DOCX) [file pone.0077998.s008.docx]

**Table S3:** P-values depicting statistical comparisons (U-test) of odorant-induced currents recorded from functionally expressed rat and human TRP channels. In: inwardly directed currents at -100 mV, out: outwardly directed currents at +100 mV.

|  | **TRPV1_in_** | **TRPV1_out_** | **TRPM8_in_** | **TRPM8_out_** | **TRPA1_in_** | **TRPA1_out_** |
| --- | --- | --- | --- | --- | --- | --- |
| **vanillin** | p=5.54*10^-3^ | p=0.12 | p=0.0167 | p=0.08 | p=0.44 | p=0.38 |
| **HTPA** | p=3.78*10^-5^ | p=0.88 | p=0.74 | p=0.17 | p=2.5*10^-4^ | p=0.6 |
| **helional** | p=3.78*10^-5^ | p=0.0723 | p=0.18 | p=0.28 | p=0.74 | p=0.6 |
| **geraniol** | p=5.72*10^-4^ | p=0.1146 | p=0.37 | p=0.28 | p=0.06 | p=0.6 |
